# TGFBR1 gene silencing attenuates cardiomyopathy in the HFpEF mouse model

**DOI:** 10.1371/journal.pone.0328981

**Published:** 2025-08-29

**Authors:** Xuanyang Shen, Weidong Li, Xiaolu Jiang, Hongfu Wen, Yuan Shen, Wen Zhang, Yongjian Peng, Guoping Kang

**Affiliations:** 1 Department of Emergency, Affiliated Hospital of North Sichuan Medical College, Nanchong, China; 2 Science and Technology Innovation Center of North Sichuan Medical College, Nanchong, China; 3 Department of Emergency, Bazhong central hospital, Bazhong, China; 4 Department of Clinical Medicine, North Sichuan Medical College, Nanchong, China; Emory University School of Medicine, UNITED STATES OF AMERICA

## Abstract

Myocardial fibrosis is a principal factor in the progression of heart failure with preserved ejection fraction (HFpEF). Prior research has substantiated the profibrotic impact of transforming growth factor β1. Nevertheless, the function of the transforming growth factor beta receptor (TGFBR) in HFpEF remains uncertain. In this study, we found an increase in myocardial TGFBR1 expression in mice with HFpEF. Silencing of the TGFBR1 gene improved cardiac function in HFpEF mice by attenuating cardiac fibrosis, reducing myocardial hypertrophy, and ameliorating myocardial remodeling. At the mechanistic level, TGFBR1 gene silencing led to a reduction in myocardial collagen synthesis through the Smad2/3 signaling pathway and an inhibition of cardiac hypertrophy through the mitogen-activated protein kinase (MAPK) signaling pathway in HFpEF mice. Additionally, we discovered that TGFBR1 gene silencing mitigated myocardial remodeling in HFpEF mice by suppressing TAK1-mediated PANoptosis, primarily because TGFBR1 gene silencing impeded the dissociation of RIPK1 and TAK1 and reduced Tumor Necrosis Factor Receptor-Associated Factor 6 expression. These findings indicate that TGFBR1 gene silencing enhances cardiac remodeling and function and represents a potential therapeutic target for the treatment of HFpEF.

## 1. Introduction

Heart failure (HF) emerges as a major public health issue of global concern, posing significant health risks to millions of people worldwide [1]. In 40–50% of HF patients, there is no reduction in left-ventricular ejection fraction (LVEF), a condition known as HF with preserved ejection fraction (HFpEF) [2–4]. The incidence and prevalence of HFpEF have increased in recent years, driven by increasing metabolic disorders such as obesity and diabetes [4–6]. Although the mortality rate associated with HFpEF may be lower than that of HF with reduced ejection fraction (HFrEF), patients experience a high rate of rehospitalization and a poor quality of life [5,7]. There is a lack of effective therapeutic agents for HFpEF, and its pathogenesis is not yet fully understood [8,9]. Consequently, it is crucial to investigate the pathological mechanisms underpinning HFpEF and to identify the potential therapeutic targets.

The primary manifestations of HFpEF include diastolic dysfunction, exercise intolerance, and pulmonary congestion [10,11]. The pathogenesis of HFpEF is primarily triggered by metabolic disturbances that lead to mitochondrial dysfunction, oxidative stress [12,13], and impaired cellular function [14]. A crucial aspect of HFpEF progression involves a systemic proinflammatory state induced by comorbidities, which produces a range of inflammatory cytokines that may exacerbate HFpEF [15]. Notably, transforming growth factor β1 (TGF-β1) is elevated in the myocardium of patients with HFpEF and has been implicated in its development [16]. TGF-β1 binds to the type II TGFβ receptor (TGFBR2) on the cell membrane, which then phosphorylates and activates TGFBR1. Subsequently, TGFBR1 transmits signals to the nucleus via Smad proteins, playing a key role in the regulation of genes involved in cell differentiation, proliferation, and apoptosis [17].

TGFBR1 also interacts with the X-linked inhibitor of apoptosis, which in turn binds to TAB1 [18]—the primary binding protein of transforming growth factor-β–activated kinase-1 (TAK1)—thereby linking TAK1 to TGFBR1 indirectly. Additionally, TAK1 directly binds to the cytoplasmic structural domain of TGFBR2, forming a TGFβ receptor protein complex at the cell membrane [19]. Upon exposure to TGFβ, the interaction between TGFBR2 and TAK1 rapidly activates TAK1 through the TGFβ receptor complex. Once activated, TAK1 phosphorylates mitogen-activated protein kinase kinases 3/6 (MAPK3/6), leading to a series of physiopathological responses [20].

Programmed cell death (PCD) is a genetically controlled process of active cell death, which mainly includes three pathways: cell pyroptosis, apoptosis and necroptosis [21–23]. Previous studies suggested that the three pathways of PCD are independent, whereas recent studies have revealed a wide range of interactions between pyroptosis and apoptosis and necrotic apoptosis. Several pathogenic factors, such as Influenza A virus (IAV) infection [24,25], inhibition of homeostasis-maintaining TAK1 [26], and altered function of receptor-interacting protein kinase 1 (RIPK1) [27], can simultaneously induce pyroptosis, apoptosis, and necroptosis in cells, and this outcome cannot be altered by inhibition of a single PCD pathway, a phenomenon known as PANoptosis [25,28,29]. The activation and execution of PANoptosis are controlled by TAK1, ZBP1, AIM2 and the PANoptosome complex, which includes NLRP3, caspase-8, RIPK1, and other components of the PCD pathway [30–32]. The PANoptosome complex consists of key molecules of the PANoptosis pathway, MLRP3, ASC, caspase8 and RIPK1, among others [28–32].

TAK1, a key member of the MAPK3 family with serine/threonine protein kinase activity, serves as an essential regulator of the nuclear factor kappa-B (NF-κB) signaling pathway and plays a crucial role in inflammatory gene expression [33]. It is also a pivotal kinase in signaling through the tumor necrosis factor-α (TNFα) receptor and the interleukin-1 (IL-1) receptor [33,34]. Under physiological conditions, TAK1 forms stable complexes with receptor-interacting protein kinase 1 (RIPK1) at these receptor proteins [35,36]. However, under certain pathological circumstances, the homeostasis between RIPK1 and TAK1 is disrupted. The disruption allows RIPK1 to recruit additional molecules, activating all three pathways of programmed cell death (PCD) concurrently, which is known as TAK1-mediated PANoptosis; the activation of PANoptosis could not be suppressed by inhibiting a single pathway of PCD [26,28,37]. While myocardial PCD has extensively been studied in various heart diseases, the mechanisms underlying TAK1-mediated PANoptosis in HFpEF remain less well characterized [38–40].

In summary, TGF-β1 correlates with HFpEF, and TGF-β1 can activate TAK1 through the interaction with TGFBR1 and TGFBR2. Moreover, TAK1 plays a significant role in myocardial remodeling and fibrosis [40]. However, the roles and mechanisms of TGFBR1, TGFBR2, and TAK1 in HFpEF are less clear. Here, we hypothesized that TGFBR1 or TGFBR2 may improve cardiac function in HFpEF mice by modulating the TAK1 signaling pathway, and we designed and conducted a series of in vivo and in vitro experiments to validate this hypothesis.

## 2. Materials and methods

### 2.1. Construction and delivery of AAV9

A type 9 AAV system (AAV9) carrying scramble shRNA (Scr) and TGFBR1 shRNA (shTGFBR1) was constructed by Obiosh (Shanghai, CN) and applied to silence the cardiac expression of TGFBR1.The following sequences of the shRNAs were used: TGFBR1, the sense sequences: 5’-GCTGACAGCTTTGCGAATTAA-3, negative control, the sense sequences: 5’-GAAGTCGTGAGAAGTAGAA −3.

### 2.2. Experimental animals

The experimental procedures used in this work were approved by the Ethics Committee of North Sichuan Medical College (Approval Code: 2023107; Approval Date: 11-17-2023) and complied with the Animal Research: Reporting of In Vivo Experiments guidelines. Eight-week-old male C57BL/6 N mice (20–24 g, pathogen-free grade) were obtained from the Animal Centre of North Sichuan Medical College. The mice were confined in cages, with unrestricted free access to water and food. The cages were placed in a well-ventilated room. The room temperature was 24 ± 2°C, the humidity was 50% ± 15% throughout the experimental period, and the light/dark cycle was 12 hours.

Twenty-four C57BL/6 N mice were randomly assigned to four groups (each consisting of six mice), namely the control, shTGFBR1 + control, HFpEF, and shTGFBR1 + HFpEF groups. The control group was provided with a standard basal diet. The shTGFBR1 + control group received the basal diet along with a tail-vein injection of AAV9-shTGFBR1 (4 × 10^11^ vg). The HFpEF group was subjected to a high-fat diet (HFD, Xiaoshu Youtai), with 60% of their caloric intake derived from fats, in conjunction with Nω-Nitro-L-arginine methyl ester (L-NAME, 0.5 g/L, HY-18729A, Sigma-Aldrich) in their drinking water [41]. The shTGFBR1 + HFpEF group was fed the HFD with L-NAME and received a tail-vein injection of AAV9-shTGFBR1 (4 × 10^11^ vg).

After 8 weeks, the mice were euthanized by an intraperitoneal injection of pentobarbital sodium (150 mg/kg, P3761 Sigma-Aldrich), and the heart was removed for further analysis.

### 2.3. Cell culture

H9C2 cells (Sichuan Bio Biotech Co., CN) were used for in vitro experimental studies. They were inoculated in DMEM/F12 (C3132, XPBioMed) dishes containing 10% FBS (C2880, XPBioMed) and cultured in 5% CO_2_ at 37°C. The cells were transfected with si-RNA using Lipofectamine^TM^ 2000 (11668019, Thermo Fisher Co.) following the manufacturer’s instructions, and subsequent treatments were performed 6 hours after the transfection. H9C2 cells were simultaneously exposed to palmitate (PA; 0.2 mM, P0500, Sigma-Aldrich) and high glucose (HG; 12.5 mM) for 12 hours as a “double-damage” cellular model, which mimics an HFpEF-like diastolic dysfunction phenotype similar to HFpEF [13].

To explore the role of TGFBR1 in the development of HFpEF, H9C2 cells were divided into the following two groups: the control group, with low-glucose (5.5 mM) treatment; the PA + HG group, with PA + HG treatment.

To explore whether silencing TGFBR1 could inhibit PA and HG–induced H9C2 cells PANoptosis, H9C2 cells were divided into the following seven groups: the control group, with low-glucose treatment; the PA + HG group, with PA + HG treatment; the si-TGFBR1 + PA + HG group, with siRNA silencing of TGFBR1 and PA + HG treatment; the si-RIPK1 + PA + HG group, with siRNA silencing of RIPK1 and PA + HG treatment; the si-NLRP3 + PA + HG group, with siRNA silencing of NLRP3 and PA + HG treatment; the si-caspase-8 + PA + HG group, with siRNA silencing of caspase-8 and PA + HG treatment; and the si-RIPK1 + NLRP3 + caspase-8 + PA + HG group, with siRNA silencing of RIPK1 + NLRP3 + caspase-8 and PA + HG treatment.

To explore whether TGFBR1 could inhibit PA and HG–induced cardiac hypertrophy, H9C2 cells were divided into the following three groups: the control group, with low-glucose treatment; the PA + HG group with PA + HG treatment; and the siTGFBR1 + PA + HG group, with siRNA silencing of TGFBR1 and PA + HG treatment.

To explore whether TGFBR1 kinase activity was necessary for TAK1 activation, H9C2 cells were divided into the following four groups: the control group, with low-glucose treatment; the SB505124 group, with SB505124 (10 μM) treatment [42]; the PA + HG group, with PA + HG treatment; and the SB505124 + PA + HG group, with PA + HG + SB505124 treatment.

To explore the effect of silencing Tumor Necrosis Factor Receptor-Associated Factor 6 (TRAF6) on TAK1 activation, H9C2 cells were divided into the following four groups: the control group, with low-glucose treatment; the si-TRAF6 group, with siRNA silencing of TRAF6; the PA + HG group, with PA + HG treatment; and the siTRAF6 + PA + HG group, with siRNA silencing of TRAF6 and PA + HG treatment.

To explore the effect of silencing TGFBR1 on the expression of TRAF6, H9C2 cells were divided into the following four groups: the control group, with low-glucose treatment; si-TGFBR1 group, with siRNA silencing of TGFBR1 treatment; the PA + HG group,with PA + HG treatment; si-TGFBR1 + PA + HG group: siRNA silencing of TGFBR1 and PA + HG treatment.

### 2.4. Echocardiography

After the mice had been anesthetized with 2% isoflurane (0.3–0.5 L/minute), cardiac geometry and function were measured using an echocardiograph (Mindray, CN). Doppler echocardiography was used to measure the left-ventricular end-diastolic diameter (LVEDD), the left-ventricular end-systolic diameter (LVESD), the interventricular septal thickness in diastole (IVS, d), the left-ventricular ejection fraction (LVEF), the ratio of peak early mitral diastolic velocity to peak late mitral diastolic velocity (E/A), the ratio of early mitral diastolic velocity to early diastolic velocity (E/E´), and the global longitudinal strain (GLS) of the left ventricle. We measured three consecutive cardiac cycles and calculated the mean values for the listed parameters [43].

### 2.5. Exercise exhaustion test

After 3 days of acclimatization to treadmill exercise, an exhaustion test was performed in the experimental groups of mice. The mice ran uphill (20°) on the treadmill (Columbus Instruments) starting at a warm-up speed of 5 m/minute for 4 minutes, after which the speed was increased to 14 m/minute for 2 minutes. Every subsequent 2 minutes, the speed was increased by 2 m/minute until the mouse was exhausted. Exhaustion was defined as the inability of the mouse to return to running within 10 seconds of direct contact with an electric-stimulus grid. Running time was measured, and running distance was calculated [13].

### 2.6. Histology and immunohistochemistry (IHC)

The left ventricle of the mice were isolated, fixed in 4% paraformaldehyde, embedded in paraffin, and cut into 5-μm-thick cross-sections. H&E, WGA, and Masson’s trichrome staining were performed following the manufacturer’s instructions using appropriate staining kits (G1120, G1346 Solarbio and L4895, Sigma-Aldrich). For IHC, the sections were dewaxed and subjected to antigen retrieval with citrate buffer (pH 6.0). The sections were then blocked with 5% goat serum for 30 minutes at 37°C and incubated with primary antibodies at 4°C overnight. The next day, the sections were incubated with horseradish peroxidase (HRP)-conjugated secondary antibodies (FNSA-0004, Fine Teast-Bio) for 30 minutes at room temperature, and detection was performed using a 3-diaminobenzidine (DAB) kit (KIT-5010, Maxim-Bio) [44]. Hematoxylin was used for nuclear staining. All histological images were examined and photographed under a microscope and analyzed with the Image J 9.0 software (Image J, USA). The antibodies used for IHC analysis were as follows: collagen I (1:200, AF7001, Affbiotech), collagen III (1:200, AF0135, Affbiotech), p-TAK1 (1:200, AF4379, Affbiotech), and TRAF6 (1:200, AF5376, Affbiotech).

### 2.7. Immunofluorescence staining

For immunofluorescence staining, the cells were first seeded on glass coverslips in the corresponding plates. Next, they were fixed in 4% paraformaldehyde for 15 minutes, permeabilized in 0.1%Triton X-100 in phosphate-buffered saline (PBS) for 5 nonblocked with 5% BSA, and incubated with primary antibodies (Affbiotech-Bio) at 4°C overnight. On the next day, the cell coverslips were incubated with Alexa Fluor 488/594–conjugated secondary antibodies (Affbiotech-Bio). The cell nuclei were stained with DAPI, which were observed and photographed using a fluorescence microscope. The following antibodies were used for immunofluorescence analysis: TGFBR1 (1:1000, AF4379, Affbiotech) and TRAF6 (1:1000, AF5376, Affbiotech).

### 2.8. Tail-cuff blood pressure recordings

Noninvasive blood pressure measurements were conducted on conscious mice using the CODA instrument (Kent Scientific) and the tail-sleeve method. Before the assessment, the mice were acclimatized to short-term restraints by being placed on a temperature-controlled platform (at 37°C) for 3 days. Blood pressure values were measured three times a day for 3 consecutive days in a quiet environment. The average of the nine blood pressure values recorded over the 3 days for each mouse was then statistically analyzed [41].

### 2.9. TUNEL staining

The mouse left ventricle tissue sections were deparaffinized and treated with proteinase K (20 µg/mL) for 30 minutes at 37°C for antigen repair following the One Step TUNEL Apoptosis Detection Kit (S1086, Beyotime) protocol. TdT buffer was prepared in line with the kit’s instructions and added dropwise to the sections, and DAPI was used to stain the nuclei of the cells after the incubation was completed. All histological images were examined and photographed under a microscope, and the proportion of TUNEL-positive cells was analyzed using Image 9.0 software.

### 2.10. Co-immunoprecipitation (Co-IP)

Co-IP assays were performed using the Co-IP kit (P2177M, Beyotime) following the manufacturer’s instructions. Myocardial tissues were lysed with RIPA buffer with added protease and phosphatase inhibitors for 30 minutes on ice. After centrifugation at 12,000 rpm for 15 minutes, 100 µL of the supernatant was transferred to a new tube, and 1 μg of primary antibody (Affbiotech-Bio) or IgG was added and incubated at 4°C for 1 hour. Protein A/G-agarose beads were then incubated with the mixture at 4°C overnight. The next day, the magnetic beads were washed five times by centrifugation of the lysate, and the immunoprecipitated proteins were incubated with the loading buffer at 100°C for 10 minutes before being used for western blot analysis. The following antibodies were used for Co-IP analysis: TGFBR1 (1:1000, AF4379, Affbiotech), TAK1 (1:1000, AF7616, Affbiotech), and RIPK1 (1:1000, AF7877, Affbiotech).

### 2.11. Western blot

The protein extraction method used was consistent with that of the Co-IP technique. Next, 15–30 µg of total protein was added to the top sample wells, and electrophoresis was used to separate proteins in 8–15% polyacrylamide gels. After transferring the proteins to a 0.45-μm PVDF membrane (HVLP09050, Sigma-Aldrich) in an ice bath, the membrane was incubated with 5% skimmed milk powder for 1 hour at room temperature. Primary antibodies were incubated at 4°C overnight, and secondary HRP-conjugated antibodies were incubated for 1 hour at room temperature. Images were analyzed with Image J software, and GAPDH was used as a control to verify equal protein loading. Quantitative analysis of phosphorylated proteins used their total proteins as a control to verify that protein loads were equal. The following antibodies were used for western blot analysis: TGFBR1 (1:1000, AF4379, Affbiotech), TGFBR2 (1:1000, AF5449, Affbiotech), collagen I (1:1000, AF7001, Affbiotech), collagen III (1:1000, AF0135, Affbiotech), Smad2 (1:1000, AF6449, Affbiotech), Smad3 (1:1000, AF6362, Affbiotech), p-Smad2 (1:1000, AF8314, Affbiotech), p-Smad3 (1:1000, AF8315, Affbiotech), TAK1 (1:1000, AF7616, Affbiotech), p-TAK1 (1:1000, AF4379, Affbiotech), NLRP3 (1:1000, AF4620, Affbiotech), caspase-1 (1:1000, AF5418, Affbiotech), GSDMD (1:1000, AF4012, Affbiotech), ASC (1:1000 DF6304, Affbiotech), caspase-3 (1:1000, AF6311, Affbiotech), caspase-7 (1:1000, DF6411, Affbiotech), caspase-8 (1:1000, AF6422, Affbiotech), RIPK1 (1:1000, AF7877, Affbiotech), RIPK3 (1:1000, AF7942, Affbiotech), MLKL (1:1000, DF7412, Affbiotech), p-MLKL (1:1000, AF7420, Affbiotech), ERK1/2 (1:1000, BF8004, Affbiotech), p-ERK1/2 (1:1000, AF1015, Affbiotech), JNK1/2 (1:1000, AF6318, Affbiotech), p-JNK1/2 (1:1000, AF3318, Affbiotech), p38 (1:1000, AF6456, Affbiotech), p-p38 (1:1000, AF4001, Affbiotech), MEK1/2 (1:1000, AF6385, Affbiotech), p-MEK1/2 (1:1000, AF8035, Affbiotech), TRAF6 (1:1000, AF5376, Affbiotech), and GAPDH (1:1000, AF7021, Affbiotech).

### 2.12. RT-PCR

Total RNA was extracted from H9C2 cells and mouse left ventricle tissues using the RNA extraction kit (K3101, Karroten-Bio). The reverse-transcription program was set following the instructions of the RNA reverse-transcription kit (K1072, APExBio) as follows: 25°C for 2 minutes, 45°C for 50 minutes, and 75°C for 15 minutes, converting total RNA into cDNA. The RT-PCR program was set following the instructions of the RT-PCR kit (K1070, APExBio) as follows: 95°C for 2 minutes, one cycle; 95°C for 15 seconds, 60°C for 45 seconds, 40 cycles; and 95°C for 15 seconds, 60°C for 60 seconds, one cycle. The reverse-transcription products were amplified using SYBR Green qPCR Mix (APEx-Bio) on the Light Cycler 480 instrument (Bio-RAD). All samples were repeated three times, and GAPDH was used for normalization in each experiment, with relative gene expression levels determined using the 2^−ΔΔCt^ method. The Kyoto Encyclopedia of Genes and Genomes (KEGG; https://www.kegg.jp/) was used for primer design. The following sequences of the PCR primers were used: TGFBR1, forward primer: 5’-TCTGCTCCTCTCGCTACAGT-3’, reverse primer: 5’-TCCAGCTCCTTCACCCTACA-3’; TRAF6 mouse/rat cells, forward primer: 5’-CTACCCGCTTTGACATGGGT-3’, reverse primer: 5’-GTACATGGACGGGT-3’, GAPDH mouse/rat cells: forward primer: 5’-GGCAAATTCAACGGCACAGT-3’, reverse primer: 5’-TAGGGCCTCTCTTGCTCAGT-3’.

### 2.13. siRNA and transfection

The oligonucleotides for siRNA were produced by IBSBio. The following sequences of the siRNAs were used: negative control, the sense sequences: 5’-UUCUCCGAACGUGUCACGUTT −3, the antisense sequences: 5’-ACGUGACACGUUCGGAGAATT −3,TGFBR1, the sense sequences: 5’-GGACCAUUGUGUUACAAGAAA-3, the antisense sequences: 5’-UCUUGUAACACAAUGGUCCUG-3’, NLRP3, the sense sequences: 5’-ACAAUCAGAUGUGGUAUACAG-3, the antisense sequences: 5’-GUAUACCACAUCUGAUUGUGU-3’, RIPK1, the sense sequences: 5’-UUUGAUUGCUCAGAAUCUCCA-3, the antisense sequences: 5’-GAGAUUCUGAGCAAUCAAAAU-3’, caspase-8, the sense sequences: 5’-UUGAGUUAAUCAUUAGAGGUG-3, the antisense sequences: 5’-CCUCUAAUGAUUAACUCAAAC-3’, TRAF6, the sense sequences: 5’-UUAAGAGACUCAUAGUAGCUC-3, the antisense sequences: 5’-GCUACUAUGAGUCUCUUAAAC-3’. H9C2 cells were transfected with Lipofectamine 2000 (11668019, Thermo Fisher Co.) at a concentration of 75 pM of siRNA double-stranded, and intervention experiments were performed 4 hours after the transfection. The transfection efficiency was determined using quantitative reverse transcription polymerase chain reaction (RT-qPCR) or western blot analysis.

### 2.14. Enzyme-linked immunosorbent assay (ELISA)

Levels of NT-proBNP, IL-1β, and IL-18 were measured using an ELISA kit (EM0109, Fine Teast-Bio). Samples were collected and centrifuged at 3000 rpm for 20 minutes. The concentrations of NT-proBNP, IL-1β, and IL-18 were then determined following the manufacturer’s instructions.

### 2.15. Statistical analysis

Data are presented as the mean ± SEM. The normality assumption of the data distribution was assessed using the Shapiro–Wilk test. Differences were analyzed using the two-tailed unpaired Student’s t test for the experiments with two groups and one-way ANOVA followed by Tukey’s post-hoc test for multiple comparisons in the experiments including three or more groups. A *p* value lower than 0.05 was considered statistically significant (Ns: **p >* *0.05, *p < 0.05, **p < 0.01, ***p < 0.001, and ****p < 0.0001). All statistical analyses were performed with GraphPad Prism 9.0 software (GraphPad, USA).

## 3. Results

### 3.1. TGFBR1 expression is increased in the hearts of HFpEF mice and in “double-damage” H9C2 cells

To clarify the role of TGFBR1/2 in the development of HFpEF, we established a mouse model of HFpEF by continuously administering HFD and L-NAME for 8 weeks [41]. Additionally, we exposed H9C2 cells to PA at a concentration of 0.2 mM and HG at 12.5 mM to create a “double-damage” cell model. This approach effectively mimics the diastolic dysfunction phenotype characteristic of HFpEF [13].

**Fig 1:**
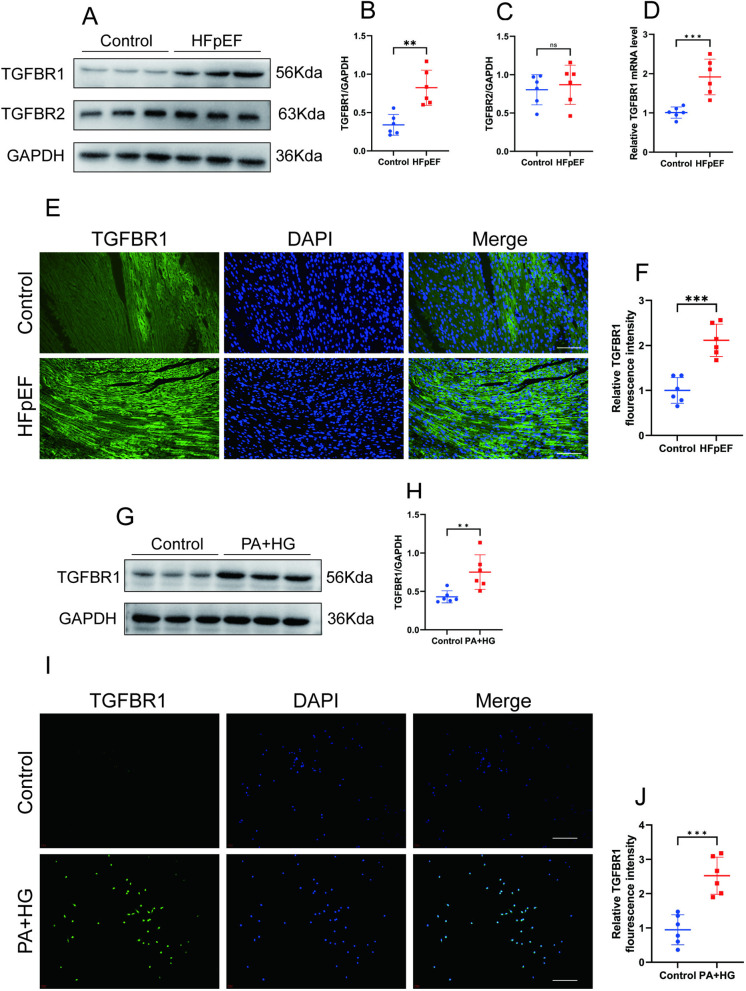
TGFBR1 expression is increased in the hearts of HFpEF mice and in “double-damage” H9C2 cells. **(A)** Representative western blot images of TGFBR1 and TGFBR2 expression in the left-ventricular samples from the control and HFpEF mice. **(B)** Quantification of the protein expression of TGFBR1 in **(A)**; n = 6 per group. **(D)** Relative mRNA levels of TGFBR1 in the left-ventricular samples from the control and HFpEF mice; n = 6 per group. **(E)** Representative immunofluorescence staining for TGFBR1 (green) in the tissue sections from the left ventricle of the control and HFpEF mice. The nuclei were counterstained with DAPI (blue). Bar = 50 μm. **(F)** Quantification of fluorescence intensity of TGFBR1 in **(E)**; n = 6 per group. **(G)** Representative western blot images of TGFBR1 expression in the groups of H9C2 cells; n = 6 per group. **(H)** Quantification of the protein expression of TGFBR1 in **(G)**. **(I)** Representative immunofluorescence staining for TGFBR1 (green) in the control and “double-damage” H9c2 cells. The nuclei were counterstained with DAPI (blue). Bar = 50 μm. **(J)** Quantification of fluorescence intensity of TGFBR1 in **(G)**; n = 6 per group. The normality of data distribution was tested using the Shapiro–Wilk method. Unpaired two-tailed Student’s t tests were applied in **(B)**, **(C)**, **(D)**, **(F)**, **(H)**, and **(J)**. *p < 0.05, **p < 0.01, and ***p < 0.001 between the indicated groups.

Immunofluorescence staining and western blot analyses demonstrated an increased expression of TGFBR1 in the myocardium of HFpEF mice compared with the control group (Fig 1A–B and E–F). In contrast, there was no significant difference in the expression of TGFBR2 between the hearts of HFpEF mice and those in the control group (Fig 1C). RT-PCR analysis further indicated that the mRNA levels of TGFBR1 were elevated in the myocardium of HFpEF mice relative to the control group (Fig 1D). Additionally, western blot analysis was performed to assess TGFBR1 expression in control and “double-damage” cell models. The results revealed a significantly higher expression of TGFBR1 in the “double-damage” cell model than in the control group (Fig 1G–H), which was corroborated by similar findings from immunofluorescence analysis (Fig 1I–J).

### 3.2. TGFBR1 gene silencing improves cardiac function in HFpEF mice

**Fig 2:**
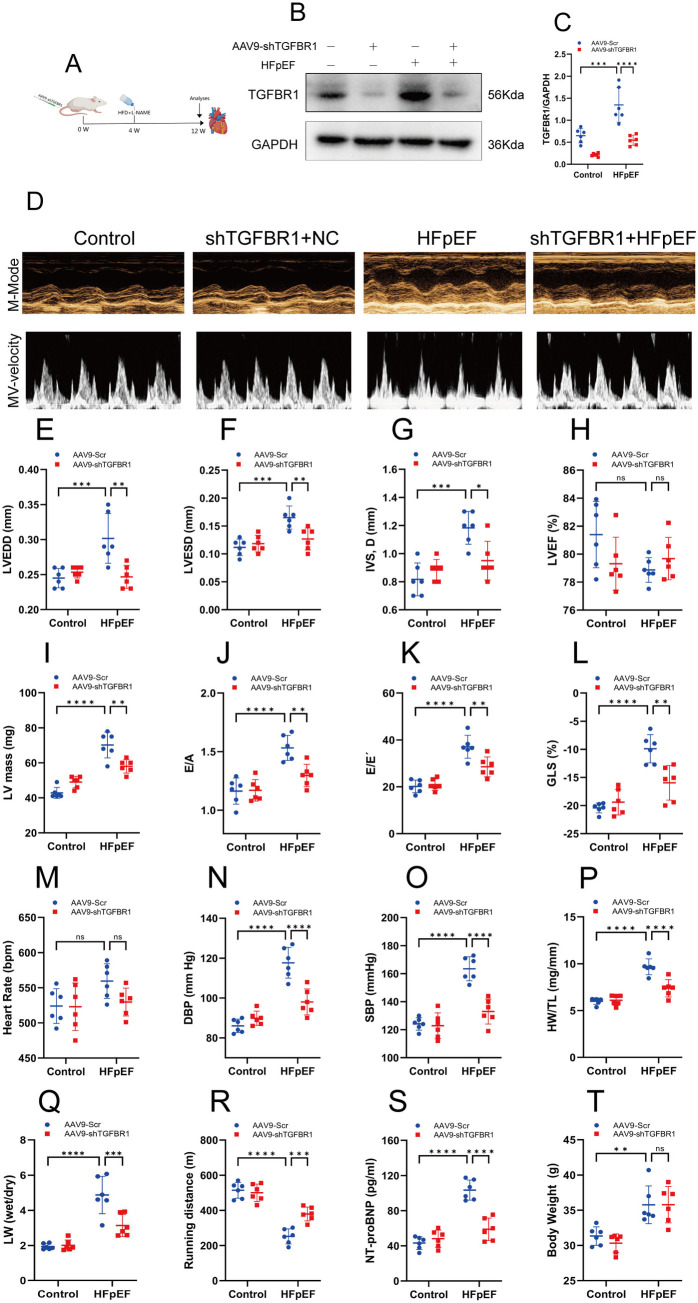
TGFBR1 gene silencing improves cardiac function in HFpEF mice. **(A)** Illustrative experimental protocol. **(B)** Representative western blot images of TGFBR1 expression in the left-ventricular samples from each group of mice. **(C)** Quantification of the protein expression of TGFBR1 in **(B)**; n = 6 per group. **(D)** Representative M-mode (upper) and pulse-wave Doppler (bottom) images. **(E)** Left-ventricular end-diastolic diameter (LVEDD). **(F)** Left-ventricular end-systolic diameter (LVESD). **(G)** Interventricular septal thickness in diastole (IVS, **d)**. **(H)** Left-ventricular ejection fraction (LVEF). **(I)** LV mass. **(J)** Ratio between mitral E wave and A wave (E/A). **(K)** Ratio between mitral E wave and E′ wave (E/E′). **(L)** Left-ventricular global longitudinal strain (GLS). **(M)** Heart rate (HR). **(N)** Diastolic blood pressure (DBP). **(O)** Systolic blood pressure (SBP). **(P)** Ratio between heart weight and tibia length (HW/TL). **(Q)** Ratio between wet and dry lung weight. **(R)** Running distance during the exercise exhaustion test. **(S)** Expression levels of N-terminal pro-B-type natriuretic peptide (NT-proBNP) in the left-ventricular samples from each group of mice. **(T)** Body weight. Mean ± SEM, n = 6 mice per group for panels E–T. The normality of data distribution was tested using the Shapiro–Wilk method. One-way ANOVA was applied in (C) and in **(E)**–(**T**). *p < 0.05, **p < 0.01, and ***p < 0.001 between the indicated groups.

To investigate the potential effects of TGFBR1 on cardiac function in HFpEF mice, we administered TGFBR1 and a control vector using AAV9 for 4 weeks, followed by the establishment of an HFpEF mouse model through continuous feeding with HFD and L-NAME for 8 weeks (Fig 2A). The results showed that TGFBR1 expression was significantly downregulated in the left ventricle of the shTGFBR1 + control and shTGFBR1 + HFpEF mice compared with the control and HFpEF groups (Fig 2B–C). Echocardiography was used to assess the effects of TGFBR1 gene silencing on cardiac function in HFpEF mice. The results indicated that TGFBR1 gene silencing effectively reversed the HFD + L-NAME–induced increases in LVEDD, LVESD, septal thickness, left-ventricular (LV) mass, E/A ratio, and E/E’ ratio, as well as reductions in the absolute value of GLS, but did not affect LVEF and heart rate(Fig 2D–M). Additionally, TGFBR1 gene silencing significantly reduced the HFD + L-NAME–induced increases in diastolic and systolic blood pressure and NT-proBNP, a marker of HF, and improved exercise intolerance, cardiac hypertrophy, and pulmonary edema (Fig 2N–R). However, it did not significantly affect body weight in HFpEF mice (Fig 2T). In contrast, empty vector administration did not replicate the beneficial effects observed with TGFBR1 silencing.

### 3.3. TGFBR1 gene silencing alleviates cardiac remodeling and fibrosis in HFpEF mice

**Fig 3:**
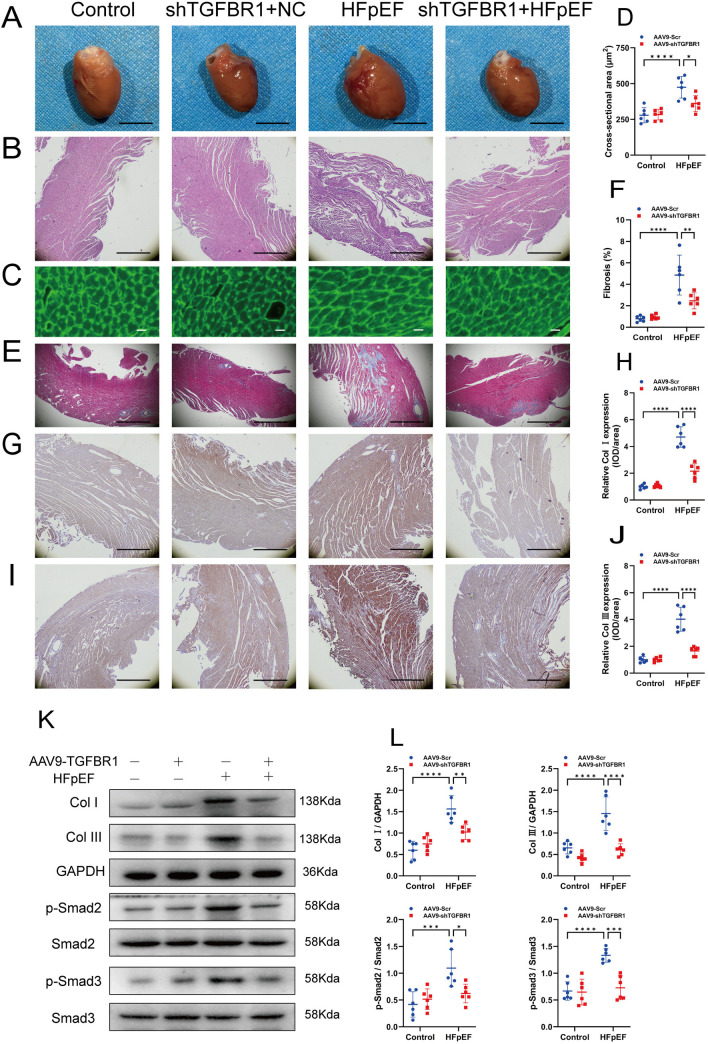
TGFBR1 gene silencing alleviates cardiac remodeling and fibrosis in HFpEF mice. **(A)** Representative images showing mouse hearts. Bar = 50 mm. **(B)** Representative hematoxylin and eosin (H&E) staining images. Bar = 200 μm. **(C)** Representative Wheat germ agglutinin (WGA) staining images. Bar = 20 μm. **(D)** Quantification cross-sectional area in **(G)**; n = 100 cells per group. **(E)** Representative Masson’s trichrome staining images. Bar = 50 μm. **(F)** Quantification percentage of interstitial fibrosis in **(E)**. **(G)** and **(I)** Representative images of myocardial type I collagen (collagen I, Col I) and type III collagen (collagen III, Col III) immunohistochemistry (IHC). Bar = 200 μm. (H) and **(J)** IHC quantification of collagen I and collagen **III. (K)** Representative western blot images of Col I, Col III, p-Smad2, and p-Smad3 expression in the left-ventricular samples from each group of mice. **(L)** Quantification of the protein expression of TGFBR1 in **(K)**. Mean ± SEM, n = 6 mice per group for panels A–L. The normality of data distribution was tested using the Shapiro–Wilk method. One-way ANOVA was applied in **(D)**, **(F)**, **(H)**, **(J)**, and **(L)**. *p < 0.05, **p < 0.01, and ***p < 0.001 between the indicated grou*p*s.

To clarify the role of TGFBR1 gene silencing in HFpEF, we analyzed its effects on cardiac hypertrophy and fibrosis. The results showed that the hearts of HFpEF mice were significantly enlarged compared with controls and that TGFBR1 gene silencing inhibited cardiac enlargement (Fig 3A). Morphological analysis revealed significant myocardial alignment disorder in the hearts of the mice from the HFpEF group, as demonstrated by H&E staining. Notably, TGFBR1 gene silencing mitigated this disorder (Fig 3B). WGA staining indicated that the cross-sectional area of left ventricular cardiomyocytes was markedly increased in the HFpEF group compared with the control group, whereas TGFBR1 gene silencing reduced the cross-sectional area of cardiomyocytes in HFpEF mice (Fig 3C–D). Additionally, Masson’s trichrome staining demonstrated an increase in the area of myocardial fibrosis in the left ventricle of HFpEF group compared with the control group, with TGFBR1 gene silencing leading to a reduction in myocardial fibrosis in HFpEF mice (Fig 3E–F). TGF-β1 is a key cytokine involved in the progression of fibrosis and is partially associated with the classical Smad signaling pathway [45]. Therefore, we assessed the expression levels of relevant mediators in the TGF-β1/Smad signaling pathway using IHC and western blot analysis. The results showed that compared with the control group, collagen I and collagen III deposition was increased in the left ventricle of HFpEF group, along with the overexpression of collagen I, collagen III, p-Smad2, and p-Smad3. Not only did TGFBR1 gene silencing reduce collagen I and collagen III deposition, but it also inhibited the overexpression of collagen I, collagen III, p-Smad2, and p-Smad3 (Fig 3G–L). These results suggest that TGFBR1 gene silencing not only inhibits myocardial remodeling but also suppresses myocardial fibrosis in HFpEF mice through the TGF-β1/Smad signaling pathway.

### 3.4. TGFBR1 gene silencing protects cardiac cells from PANoptosis-like death

TAK1 is a key kinase involved in signaling for the TNFR and IL-1R [33,40]. Under normal physiological conditions, TAK1 and RIPK1 form a stable complex with these receptor proteins [35,36]. However, under specific conditions, RIPK1 dissociates from TAK1 and recruits other molecules into the PANoptosome, thereby driving PANoptosis [26], a process referred to as TAK1-mediated PANoptosis. The assembly of the TGFBR1/2 complex activates TAK1, but it remains unclear whether TGFBR1 gene silencing can influence TAK1-mediated PANoptosis.

**Fig 4:**
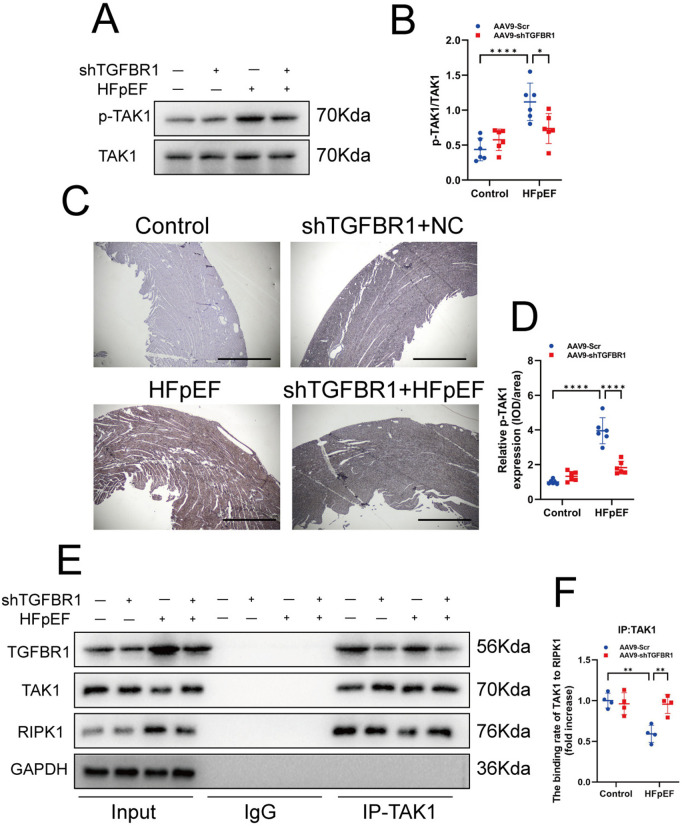
TGFBR1 gene silencing inhibits TAK1 activation and dissociation. **(A)** Representative western blot images of p-TAK1 expression in the left-ventricular samples from each group of mice. **(B)** Quantification of the protein expression of p-TAK1 in **(A)**; n = 6 per group. **(C)** Representative images of p-TAK1 immunohistochemistry (IHC). Bar = 200 μm; n = 6 per group. **(D)** Quantification of IHC of p-TAK1. **(E)** Representative immunoprecipitation images of TGFBR1, TAK1, and RIPK1. **(F)** Quantification of the binding rate of TAK1 to RIPK1; n = 4 per group. The normality of data distribution was tested using the Shapiro–Wilk method. One-way ANOVA was applied in **(B)**, **(D)**, and **(F)**. *p < 0.05, **p < 0.01, and ***p < 0.001 between the indicated groups.

To investigate the effect of TGFBR1 gene silencing on TAK1 activation, we assessed TAK1 activation across different experimental groups. The results indicated that TAK1 was activated in the left ventricle of HFpEF mice, whereas TGFBR1 gene silencing significantly inhibited TAK1 activation (Fig 4A–D). Subsequently, Co-IP was performed to analyze the interactions among TGFBR1, RIPK1, and TAK1. In the left ventricle of the control group, we observed the interaction between TAK1 and both RIPK1 and TGFBR1. However, in the left ventricle of the HFpEF group, the interaction between RIPK1 and TAK1 was significantly reduced. Importantly, TGFBR1 gene silencing restored the interaction between RIPK1 and TAK1 (Fig 4E–F).

**Fig 5:**
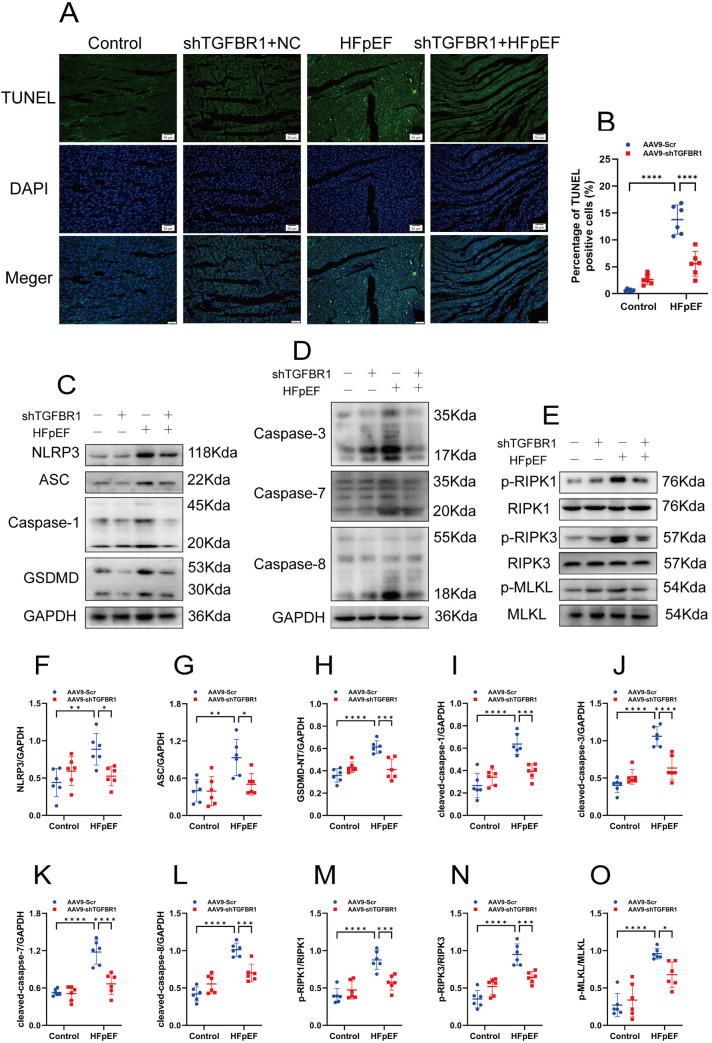
TGFBR1 gene silencing alleviates myocardial PANoptosis-like cell death in HFpEF mice. **(A)** Representative TUNEL staining images of the left-ventricular samples from each group of mice. Bar = 50 μm. **(B)** Quantification of TUNEL-positive cells in **(A)**; n = 100 cells per group. **(C)** Representative western blot images of NLRP3, caspase-1, and GSDMD expression in the left-ventricular samples from each group of mice. **(D)** Representative western blot images of caspase-3, caspase-7, and caspase-8 expression in the left-ventricular samples from each group of mice. **(E)** Representative western blot images of p-RIPK1, p-RIPK3, and p-MLKL expression in the left-ventricular samples from each group of mice. **(F)**–**(I)** Quantification of the protein expression of NLRP3, cleaved caspase-1, and GSDMD-N in **(C)**. **(J)**–**(L)** Quantification of the protein expression of cleaved caspase-3, cleaved caspase-7, and cleaved caspase-8 in **(D)**. **(M)**–**(O)** Quantification of the protein expression of p-RIPK1, p-RIPK3, and p-MLKL in **(E)**; n = 6 mice per group for panels A–O. The normality of data distribution was tested using the Shapiro–Wilk method. One-way ANOVA was applied in **(B)** and in **(F)**–**(O)**. *p < 0.05, **p < 0.01, and ***p < 0.001 between the indicated groups.

We found that the proportion of TUNEL-positive cells was significantly elevated in the myocardial tissues of HFpEF mice; however, this phenomenon was mitigated by silencing the TGFBR1 gene (Fig 5A–B). Following this, we assessed the expression of key proteins in the TAK1–PANoptosis pathway within the cardiac tissues of the mice and analyzed the effect of TGFBR1 gene silencing on these proteins. Our further investigations revealed that the following key proteins associated with the PANoptosis pathway in the myocardium of HFpEF mice were significantly upregulated: the pyroptosis-associated proteins NLRP3, cleaved caspase-1, and GSDMD-N; the apoptosis-associated proteins cleaved caspase-3, cleaved caspase-7, and cleaved caspase-8; and the necrosis-associated proteins p-RIPK1, p-RIPK3, and p-MLKL (Fig 5C–O). Notably, TGFBR1 gene silencing effectively inhibited the increases in these proteins, suggesting that it alleviates myocardial PANoptosis-like cell death in HFpEF mice.

**Fig 6:**
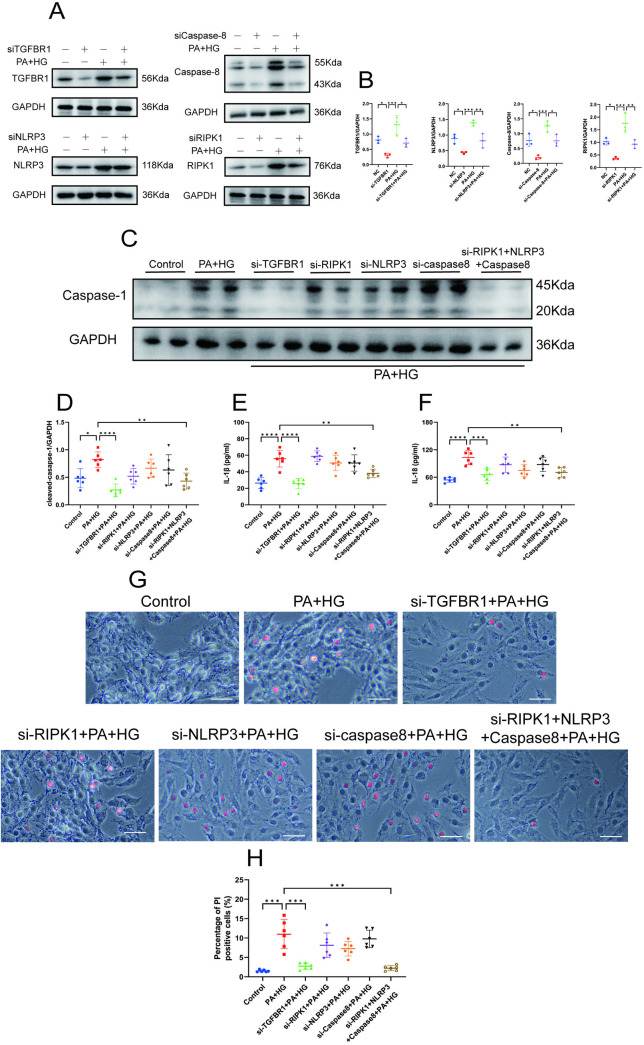
TGFBR1 gene silencing alleviates myocardial PANoptosis cell death induced by “double-damage. ” **(A)** Representative western blot images of TGFBR1, NLRP3,caspase-8 and RIPK1 expression in the groups of H9C2 cells; n = 3 per group. **(B)** Quantification of the protein expression of TGFBR1, NLRP3,caspase-8 and RIPK1. **(C)** Representative western blot images of caspase-1 expression in the groups of H9C2 cells; n = 6 per group. **(D)** Quantification of the protein expression of cleaved caspase-1 in **(C)**. **(E)** Extracellular-fluid IL-1β levels; n = 6 per group. **(F)** Extracellular-fluid IL-18 levels; n = 6 per group. **(G)** Representative PI staining images of the groups of H9C2 cells. Red indicates dead cells. Bar = 50 μm. **(H)** Quantification of PI-positive cells in **(G)**; n = 100 cells per group. The normality of data distribution was tested using the Shapiro–Wilk method. One-way ANOVA was applied in **(D)**–**(F)** and in **(H)**. *p < 0.05, **p < 0.01, and ***p < 0.001 between the indicated groups.

Moreover, the activation of PANoptosis could not be suppressed by inhibiting a single pathway of PCD. To explore this, we investigated caspase-1 activation, inflammatory factor release, and cell death by silencing the individual PCD pathways using siRNA in a “double-damage” cell model. Our results showed that transfection by siRNA effectively reduced the expression of the pyogenic pathway protein NLRP3, the apoptotic pathway protein caspase-8, the necroptosis pathway protein RIPK1, and TGFB1 in cells (Fig 6A–B). Further studies demonstrated that silencing NLRP3, caspase-8, or RIPK1 did not prevent caspase-1 activation, inflammatory factors release, or cell death in H9C2 cells. Remarkably, only silencing TGFBR1 or concurrently silencing NLRP3, caspase-8, and RIPK1 effectively rescued H9C2 cells from death induced by PA and HG (Fig 6C–H). These findings indicate that TGFBR1 gene silencing can significantly alleviate myocardial PANoptosis cell death induced by “double-damage.”

### 3.5. TGFBR1 gene silencing inhibits the assembly of PANoptosome

The activation of PANoptosis is regulated by upstream factors and the PANoptosome, a complex comprising molecules from the pyroptosis, apoptosis, and necroptosis pathways [30,25]. In TAK1-mediated PANoptosis, RIPK1 dissociates from TAK1 and moves into the intracellular compartment, where it recruits molecules from these death pathways to assemble the PANoptosome, thereby driving PANoptosis [28,37]. We found that the interaction among RIPK1, TAK1, and TGFBR1 was significantly attenuated in the myocardium of HFpEF mice and that inhibiting TGFBR1 gene expression reduced PANoptosis-like cell death in this context (see Part 3.4).

**Fig 7:**
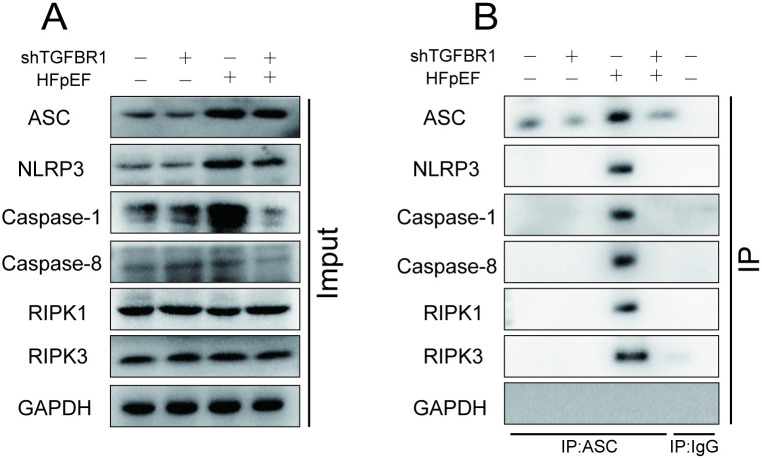
TGFBR1 gene silencing inhibits the assembly of the PANoptosome. **(A)** Representative western blot images of ASC, NLRP3, caspase-1, caspase-8, RIPK1, and RIPK3 in the left-ventricular samples from each group of mice. **(B)** Representative Co-IP images of ASC, NLRP3, caspase-1, caspase-8, RIPK1, and RIPK3 in the left-ventricular samples from each group of mice; n = 3 mice per group for panels **A** and **B.**

To investigate whether TGFBR1 gene inhibition regulates PANoptosis by suppressing the assembly of the RIPK1–PANoptosome, we used Co-IP to characterize the effect of TGFBR1 gene silencing on PANoptosome assembly in the left ventricle of HFpEF mice. In the left ventricle of HFpEF mice, interactions among NLRP3, ASC, caspase-1, caspase-8, RIPK1, and RIPK3 were markedly increased. Notably, silencing of the TGFBR1 gene suppressed these interactions (Fig 7A–B), suggesting that TGFBR1 gene silencing inhibits the assembly of the PANoptosome.

### 3.6. TGFBR1 gene silencing inhibits cardiac hypertrophy

We observed that silencing of the TGFBR1 gene inhibited cardiac hypertrophy in HFpEF mice (see Part 3.2), primarily evidenced by reduced ventricular wall thickness, heart mass, and cardiomyocyte cross-sectional area. However, the underlying mechanism remains unclear. Some studies have indicated that TGFβ1 can activate TAK1 through the TGFBR1/2 complex, with activated TAK1 promoting cardiac hypertrophy via the p38/MAPK-JNK1/2 signaling pathway [46–48]. Conversely, other investigations have shown that activation of JNK and p38 may not promote hypertrophy and could even exert an antihypertrophic effect [49,50].

**Fig 8:**
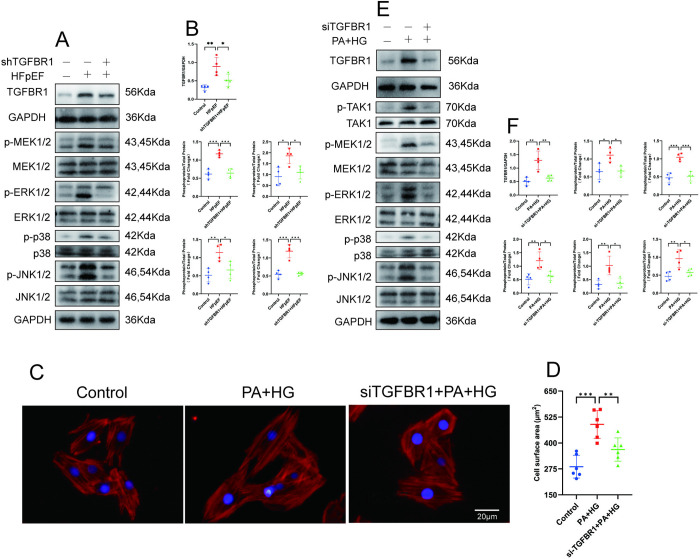
TGFBR1 gene silencing inhibits cardiac hypertrophy. **(A)** Representative western blot images of TGFBR1, p-MEK1/2, p-ERK1/2, p-p38, and p-JNK1/2 in the left-ventricular samples from each group of mice; n = 4 mice per group. **(B)** Quantification of the protein expression of TGFBR1, p-MEK1/2, p-ERK1/2, p-p38, and p-JNK1/2 in **(A)**. **(C)** Representative images of myocardial phalloidin staining. The nuclei were counterstained with DAPI (blue). Bar = 20 μm. **(D)** Quantification of cell surface area; n = 100 cells per group. **(E)** Representative western blot images of TGFBR1, p-MEK1/2, p-ERK1/2, p-p38, and p-JNK1/2 expression in the groups of H9C2 cells; n = 4 per group. **(F)** Quantification of the protein expression of TGFBR1, p-MEK1/2, p-ERK1/2, p-p38, and p-JNK1/2 in **(E)**. The normality of data distribution was tested using the Shapiro–Wilk method. One-way ANOVA was applied in **(B)**, **(D)**, and **(F)**. *p < 0.05, **p < 0.01, and ***p < 0.001 between the indicated groups.

In our study, the HFpEF mice exhibited cardiac hypertrophy along with TAK1 activation. To explore whether TGFBR1 gene silencing exerts an antihypertrophic effect through the TAK1–p38 MAPK/JNK1/2 signaling pathway, we examined the expression levels of TGFBR1, p-ERK1/2, p-JNK1/2, p-p38, and p-MEK1/2 in the left ventricle of each mouse group. Our results revealed that the expression of p-ERK1/2, p-p38, and p-MEK1/2 was elevated in the left ventricle of the HFpEF mice compared with the control group and that TGFBR1 gene silencing effectively inhibited this elevation (Fig 8A–B).

Furthermore, we found that TGFBR1 gene silencing mitigated the PA + HG–induced increase in H9C2 cells surface area in the “double-damage” cell model (Fig 8C–D). In parallel, elevated levels of TGFBR1, p-TAK1, p-ERK1/2, p-p38, and p-MEK1/2 were detected in “double-damage” H9C2 cells compared with the control group, with TGFBR1 gene silencing inhibiting their increase (Fig 8E–F). These findings suggest that TGFBR1 gene silencing may exert an antihypertrophic effect through the TAK1–p38/MAPK–JNK1/2 signaling pathway.

### 3.7. TGFBR1 gene silencing inhibits TAK1 activation via TRAF6

**Fig 9:**
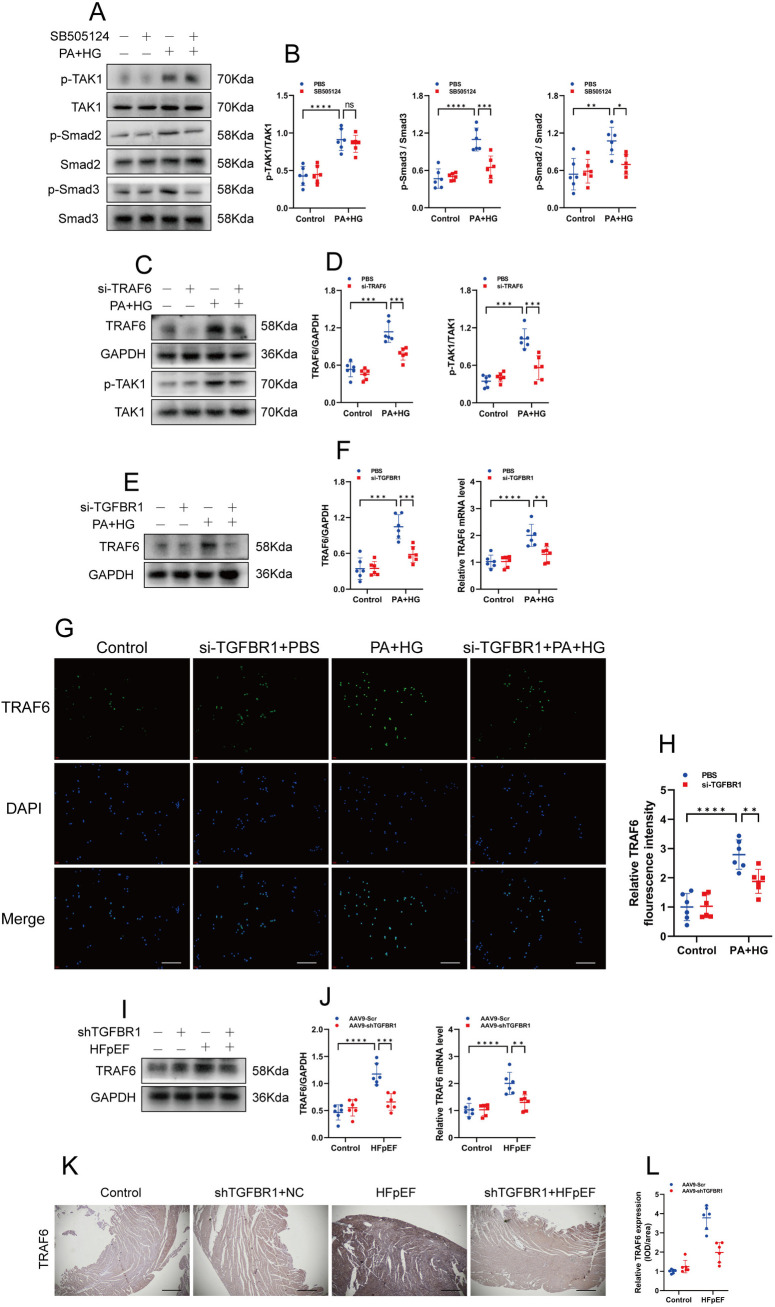
TGFBR1 gene silencing inhibits TAK1 activation via TRAF6. **(A)** Representative western blot images of p-TAK1, p-Smad2, and p-Smad3 in the groups of H9C2 cells. **(B)** Quantification of the protein expression of p-TAK1, p-Smad2, and p-Smad3 in **(A)**. **(C)** Representative western blot images of TRAF6 and p-TAK1 in the groups of H9C2 cells. **(D)** Quantification of the protein expression of TRAF6 and p-TAK1 in **(C)**. **(E)** Representative western blot images of TRAF6 in the groups of H9C2 cells. **(F)** Quantification of the protein expression of TRAF6 in **(E)**, and relative TRAF6 mRNA expression levels in the groups of H9C2 cells. **(G)** Representative immunofluorescence staining of TRAF6 (green) in the groups of H9C2 cells. The nuclei were counterstained with DAPI (blue). Bar = 50 μm. **(H)** Quantification of fluorescence intensity of TRAF6 in **(G)**; **(A)**–(H) n = 6 per group. **(I)** Representative western blot images of TRAF6 in the left-ventricular samples from each group of mice. **(J)** Quantification of the protein expression of TRAF6 in **(I)**, and relative TRAF6 mRNA expression levels in the left-ventricular samples from each group of mice. **(K)** Representative images of myocardial TRAF6 immunohistochemistry (IHC). Bar = 200 μm. **(L)** Quantification of IHC of TRAF6; n = 6 mice per group for panels I–L. The normality of data distribution was tested using the Shapiro–Wilk method. One-way ANOVA was applied in **(B)**, **(D)**, **(H)**, **(F)**, and **(L)**. *p < 0.05, **p < 0.01, and ***p < 0.001 between the indicated groups.

As mentioned previously, we have demonstrated that TGFBR1 elevation is accompanied by TAK1 activation and that TGFBR1 gene silencing inhibits TAK1 activation; however, the molecular mechanism of the inhibition remains unknown. To elucidate whether TGFBR1 kinase activity (or activin-like kinase 5, ALK5) is necessary for TAK1 activation, we used the ALK5 kinase inhibitors in “double-damage”–treated H9C2 cells. SB505124 interventions in the presence of the ALK5 kinase inhibitors did not prevent TAK1 activation, but—as expected—they prevented Smad2/3 activation (Fig 9A–B). Thus, the kinase activity of TGFBR1 is not necessary for the activation of TAK1, whereas it is necessary for the activation of Smad2. It has been shown that TRAF6 is required for TGF-β activation of the TAK1–p38/JNK pathway [51]. To verify whether TRAF6 is required for TAK1 activation in the “double-damage” cell model, we investigated TRAF6 expression and TAK1 activation status in untreated or “double-damage”–treated H9C2 cells. The results showed that TRAF6 expression was significantly increased in “double-damage”–treated H9C2 cells and that TRAF6 gene silencing inhibited TAK1 activation (Fig 9C–D). Further exploration revealed that TGFBR1 gene silencing reduced TRAF6 expression both in vivo and in vitro (Fig 9E–L). These data suggest that TGFBR1 kinase activity in “double-damage”–treated H9C2 cells is not associated with TAK1 activation and that the inhibition of TAK1 activation by TGFBR1 gene silencing is mainly attributable to the inhibition of RIPK1 and TAK1 dissociation and the reduction of TRAF6 expression.

## 4. Discussion

HFpEF is a complex and heterogeneous disease that occurs in elderly and obese patients, often accompanied by hypertension, diabetes mellitus, renal insufficiency, atrial fibrillation, and other underlying diseases, and accounts for about 50% of the hospitalizations of heart failure patients, for whom there is still a lack of effective treatment in the clinic [6,7]. The successful establishment of an animal model of HFpEF is an important foundation for the study of HFpEF pathogenesis. The animal models commonly used in the study of HFpEF are mainly pressure-loaded models, ischemic models, and composite models. Pressure-loaded HFpEF models are mainly induced by aortic constriction surgery, spontaneous hypertension rat induction, secondary hypertension rat models, or salt-sensitive rat induction, which results in an increase in cardiac afterload, cardiomyocyte hypertrophy, and fibrosis, leading to a decrease in the diastolic function of cardiomyocytes [52–55]. The ischemic HFpEF model mainly causes chronic myocardial ischemia through extracoronary chronic constriction, intracoronary chronic stenosis, and drugs, simulating HFpEF caused by chronic myocardial ischemia [53]. The composite HFpEF model, on the other hand, integrates multiple pathologic mechanisms such as hypertension, dyslipidemia, and diabetes mellitus [41,56]. Since HFpEF often coexists with multiple clinical complications, this composite model better simulates the clinicopathological features of HFpEF [13,41,56]. Although different types of HFpEF models have different pathogenesis and research targeting, the composite model unites more than two clinical factors, which are more closely related to the clinical characteristics of HFpEF and may be more representative and generalizable, representing the main developmental direction of HFpEF models. It has been shown that high glucose and HFD impair cardiac systolic and diastolic functions in mice, which are associated with the development of heart failure [57]. L-NAME is a competitive inhibitor of nitric oxide synthase, which hinders the production of the endothelial diastolic factor nitric oxide and is a classic drug for inducing hypertension in animal models. L-NAME, administered either by intraperitoneal injection or by dissolving it in drinking water, can increase the blood pressure of experimental animals, which is accompanied by significant changes in cardiac index, small artery diameter, cardiomyocyte diameter, and wall thickness [58,59]. According to a previous study [41], L-NAME was dissolved in drinking water (0.5 g/L) and fed to male mice, which were stably stimulated to maintain a constant and stable increase in blood pressure, and it was combined with HFD feeding to induce the development of HFpEF-like pathological features in mice. The results showed that mice developed reduced left ventricular diastolic function at week 8 after modeling; Doppler echocardiography demonstrated increases in LVEDD, LVESD, septal thickness, LV mass, E/A ratio, and E/E’ ratio, as well as reductions in absolute values of GLS, while not affecting LVEF. In addition, HFD + L-NAME induced elevated diastolic and systolic blood pressures, increased exercise intolerance, myocardial hypertrophy, pulmonary edema, and elevated NT-proBNP, an indicator of heart failure. The model used in this study was male mice, so these characterizations confirm that the male mouse HFpEF model established by this protocol is reliable. However, the applicability of this model to female mice requires further study.

**Fig 10:**
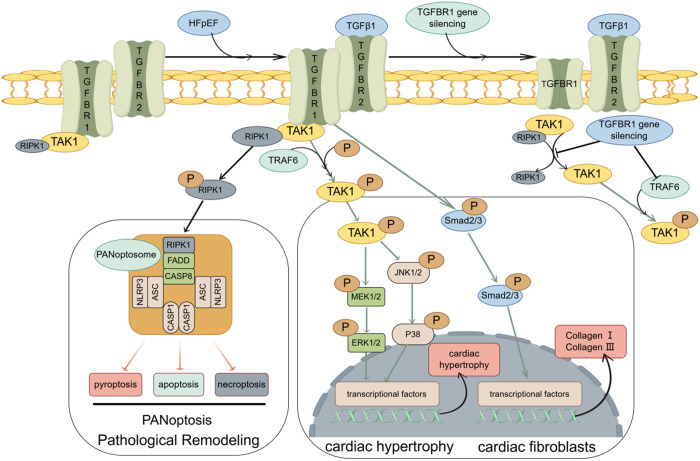
Potential mechanisms underlying the effects of TGFBR1 gene silencing on the heart of HFpEF mice are as follows. TGFBR1 gene silencing leads to a reduction in myocardial collagen synthesis through the Smad2/3 signaling pathway and inhibits myocardial hypertrophy in HFpEF mice through the p38/MAPK–JNK1/2 signaling pathway. Furthermore, TGFBR1 gene silencing impedes myocardial remodeling in HFpEF mice by curbing TAK1-mediated PANoptosis, predominantly because TGFBR1 gene silencing hinders the segregation of RIPK1 from TAK1 and reduces TRAF6 expression.

TGFBR1 is a key molecule in TGFβ signaling, which is involved in a variety of biological processes including cell differentiation, proliferation, apoptosis, and wound healing [60,61]. In cardiac diseases, TGFBR1 is mainly involved in myocardial fibrosis, and inhibition of its expression can reduce collagen deposition and improve cardiac function through the Smad signaling pathway [60,61]. In addition, it has been found that blocking TGFBR1 stimulates autocrine and paracrine secretion of survivin in Cardiac progenitor cells, promotes damage repair in the heart, and improves myocardial remodeling [62]. In the progression of pulmonary hypertension, inhibition of TGFBR1 significantly attenuates the development of pulmonary hypertension and reduces pulmonary vascular remodeling, and its effects are associated with a reduction in early vascular cell apoptosis, pericyte proliferation, and matrix metalloproteinase expression [63]. Notably, in myocardial remodeling, although inhibition of TGFβ prevented myocardial fibrosis, it did not ameliorate cardiac dysfunction, and when knockdown of TGFBR2 not only prevented myocardial fibrosis but also ameliorated cardiac dysfunction [64], and it remains unclear whether knockdown of TGFBR1 has a similar effect. Although TGFBR1 has been extensively studied in cardiovascular diseases, its role and mechanism in HFpEF remain unclear. Our study revealed that TGFBR1 expression was upregulated in the hearts of HFpEF mice as well as in PA- and HG-treated H9C2 cells, and silencing of TGFBR1 alleviated HFpEF-induced cardiac remodeling, pulmonary edema, diastolic dysfunction, exercise intolerance, and increased blood pressure but had little effect on the body weight of HFpEF mice. Mechanistically, we found that TGFBR1 gene silencing decreased myocardial collagen synthesis through the Smad2/3 signaling pathway and inhibited cardiac hypertrophy through the p38/MAPK–JNK1/2 signaling pathway in HFpEF mice. Finally, we found that TGFBR1 gene silencing improved myocardial remodeling in HFpEF mice by inhibiting TAK1-mediated PANoptosis, which was mechanistically attributed mainly to the fact that TGFBR1 gene silencing inhibited the dissociation of RIPK1 and TAK1 and decreased TRAF6 expression (Fig 10).

It has been shown that patients with HFpEF have varying degrees of diffuse interstitial myocardial fibrosis [65,66]. In addition, the degree of interstitial myocardial fibrosis correlates with poor prognosis in patients with HFpEF [67,68]. A large population-based cohort study has shown that blood biomarkers of fibrosis are associated with HFpEF but not with HFrEF [14,69]. These studies suggest that myocardial fibrosis is an important factor in the progression of HFpEF [14]. In HFpEF, comorbidity-driven myocardial inflammation is a key factor leading to myocardial tissue fibrosis [14]. Chronic inflammation induces monocytes to infiltrate damaged microvascular endothelial cells and release large amounts of TGF-β, inducing fibroblasts and myofibroblasts to proliferate and differentiate while secreting excessive amounts of collagen I, which leads to diastolic dysfunction of the heart [16,70]. In the present study, we found significant fibrosis in the cardiac tissue of HFpEF mice using Masson’s trichrome staining. By IHC staining, we observed large amounts of collagen I and collagen III deposits in the hearts of HFpEF mice. The production of collagen I and collagen III is mainly caused by TGF-β stimulation of TGFBR1/2, which subsequently activates the Smad signaling pathway in the pericardium [45]. In this study, we found that TGFBR1 was significantly elevated in the myocardium of HFpEF mice and that the Smad signaling pathway was activated. Silencing TGFBR1 inhibited this activation, reduced the expression of collagen I and collagen III, and ameliorated myocardial fibrosis in HFpEF mice.

PCD is a genetically controlled process of active cell death, which consists of three main pathways, namely cellular pyroptosis, apoptosis, and necrosis [21–23]. PCD is a key innate immune response, and its process is capable of limiting the spread of infections and removing cells in the presence of homeopathic damages, which is essential for the development of the organism and for maintaining homeostasis [71]. Although cytokinesis PCD is to some extent capable of limiting the spread of infection as well as removing cells with homologous destruction, when PCD is overactivated, such as when PANoptosis occurs, this leads to an inability to recover cellular function or even death, ultimately inducing the onset of disease. Previous studies have shown that pyroptosis, apoptosis, and necrosis are all associated with the progression of HFpEF and that inhibition of their development can improve the phenotype of HFpEF [13,56,72]; however, PANoptosis has less frequently been reported in HFpEF. In the present study, we found that all three pathways of myocardial PANoptosis were activated and that myocardial injury occurred in HFpEF mice; notably, TGFBR1 gene silencing inhibited myocardial PANoptosis-like death.

TGFBR1 is related to TAK1, and TAK1 is a key molecule in the activation of PANoptosis, which in a RIPK1-dependent manner blocks spontaneous NLRP3 inflammasome activation as well as PANoptosis [26]. When TAK1 is inhibited or activated, RIPK1 acts as a scaffold to form a complex with caspases, which then binds to ASC and NLRP3 to form the PANoptosome, thereby activating PANoptosis [26]. Our study revealed that the activation of TAK1 in the myocardium of HFpEF mice resulted in a dissociation of RIPK1 from TAK1, followed by recruitment of key PCD molecules by RIPK1 to assemble the PANoptosome, thereby activating PANoptosis and leading to myocardial injury, which was inhibited by silencing TGFBR1. The activation of PANoptosis could not be suppressed by inhibiting a single pathway of PCD. To explore this, we investigated caspase-1 activation, inflammatory factor release, and cell death by silencing the individual PCD pathways using siRNA in a “double-damage” cell model. Our results demonstrated that silencing the pyroptosis pathway protein NLRP3, the apoptotic pathway protein caspase-8, or the necroptosis pathway protein RIPK1 did not prevent caspase-1 activation, cytokine release, or cell death in H9C2 cells. Remarkably, only silencing TGFBR1 or concurrently silencing NLRP3, caspase-8, and RIPK1 effectively rescued H9C2 cells from death induced by PA and HG. These data suggest that the protective effect of TGFBR1 gene silencing on myocardial injury is mainly to inhibit myocardial PANoptosis induced by dissociation of RIPK1 from TAK1. This finding not only expands the field of research on TAK1–PANoptosis but also discovers the mechanism by which TGFBR1, TAK1, and RIPK1 regulate PANoptosis.

Some clinical studies have found that left-ventricular hypertrophy is present in 30–60% of patients with HFpEF, and although this feature is not a necessary characteristic of HFpEF, it is still an important part of the development of HFpEF [73–77]. In HFpEF, myocardial hypertrophy is often accompanied by myocardial fibrosis [78,79]. Silencing TGFBR1 attenuates myocardial fibrosis, while it is unknown whether it inhibits cardiac hypertrophy. In the present study, we found that silencing TGFBR1 inhibited myocardial hypertrophy both in vivo and in vitro. TGF-β can activate TAK1 through the TGFBR1/2 complex, and activated TAK1 can promote myocardial hypertrophy through the p38/MAPK–JNK1/2 signaling pathway [46–48]. However, some researchers have also found that activation of JNK and p38 does not promote hypertrophy or even exerts an antihypertrophic effect [49,50]. In the present study, we found that TGFBR1 gene silencing inhibited cardiac hypertrophy in HFpEF mice by suppressing the TAK1–P38/MAPK–JNK1/2 signaling pathway, and similar results were observed in vitro. These findings confirm the role of TGFBR1 gene silencing in the fight against cardiac hypertrophy and its value as a potential target against cardiac hypertrophy.

Activation of substrates by TGFBR1 primarily involves ALK5, while the promotion of HFpEF by TGFBR1 largely depends on the activation of TAK1. In this study, we found that although inhibiting TGFBR1 kinase activity reduced Smad2/3 activation, it did not activate TAK1. This indicates that TAK1 activation by TGFBR1 is not contingent upon the kinase activity of TGFBR1. Previous research has demonstrated that reactive oxygen species generated during cardiac hypertrophy progression can induce the auto-ubiquitination of TRAF6, leading to the recruitment of TAB2 and the subsequent binding to TAK1, thereby activating the TAK1-mediated prohypertrophic signaling pathway [80]. However, the role of TRAF6 in TGFBR1 regulation of TAK1-mediated cardiac hypertrophy and PANoptosis remains unclear. In our study, we observed that TRAF6 gene silencing inhibited TAK1 activation, and further investigation indicated that TGFBR1 gene silencing suppressed TRAF6 expression in the myocardium of HFpEF mice. Overall, the beneficial effects of TGFBR1 gene silencing on ameliorating myocardial remodeling in HFpEF mice primarily result from the inhibition of TAK1-mediated PANoptosis and cardiac hypertrophy. This occurs through the disruption of the interaction between RIPK1 and TAK1, as well as the reduction of TRAF6 expression.

In conclusion, we provide evidence that TGFBR1 gene silencing inhibits myocardial fibrosis, myocardial hypertrophy, and myocardial remodeling and improves cardiac function in HFpEF mice, possibly through the modulation of the Smad2/3 fibrosis pathway as well as the TAK1-mediated signaling pathways of PANoptosis and cardiac hypertrophy. This provides a research direction for TGFBR1 as a therapeutic target for HFpEF.

## Supporting information

S1 File Original image of WB. (PDF)

S2 File Figure 1 original data. (ZIP)

S3 File Figure 2 original data. (ZIP)

S4 File Figure 3 original data. (ZIP)

S5 File Figure 4 original data. (ZIP)

S6 File Figure 5 original data. (ZIP)

S7 File Figure 6 original data. (ZIP)

S8 File Figure 7 original data. (ZIP)

S9 File Figure 8 original data. (ZIP)

S10 File Figure 9 original data. (ZIP)
