# Supplementary material for: TGFBR1 gene silencing attenuates cardiomyopathy in the HFpEF mouse model
Source: PLoS One. 2025 Aug 29;20(8):e0328981. doi: 10.1371/journal.pone.0328981 (PMC12396675; doi:10.1371/journal.pone.0328981)

Original image of WB

Figure 1A Original Images for Blots

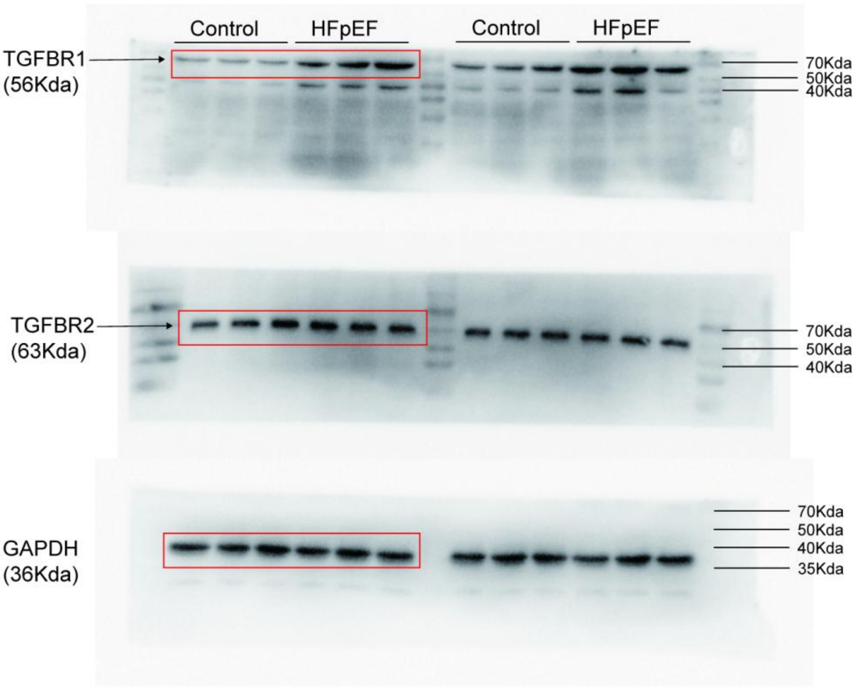

Figure 1G Original Images for Blots

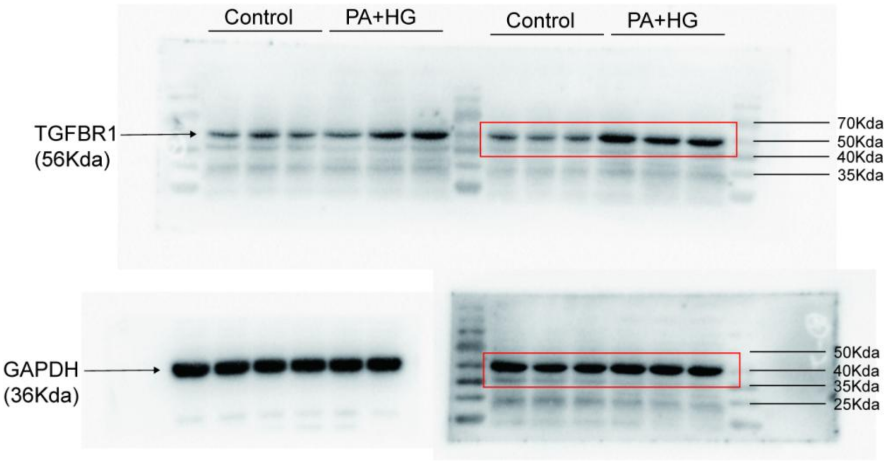

Figure 2B Original Images for Blots

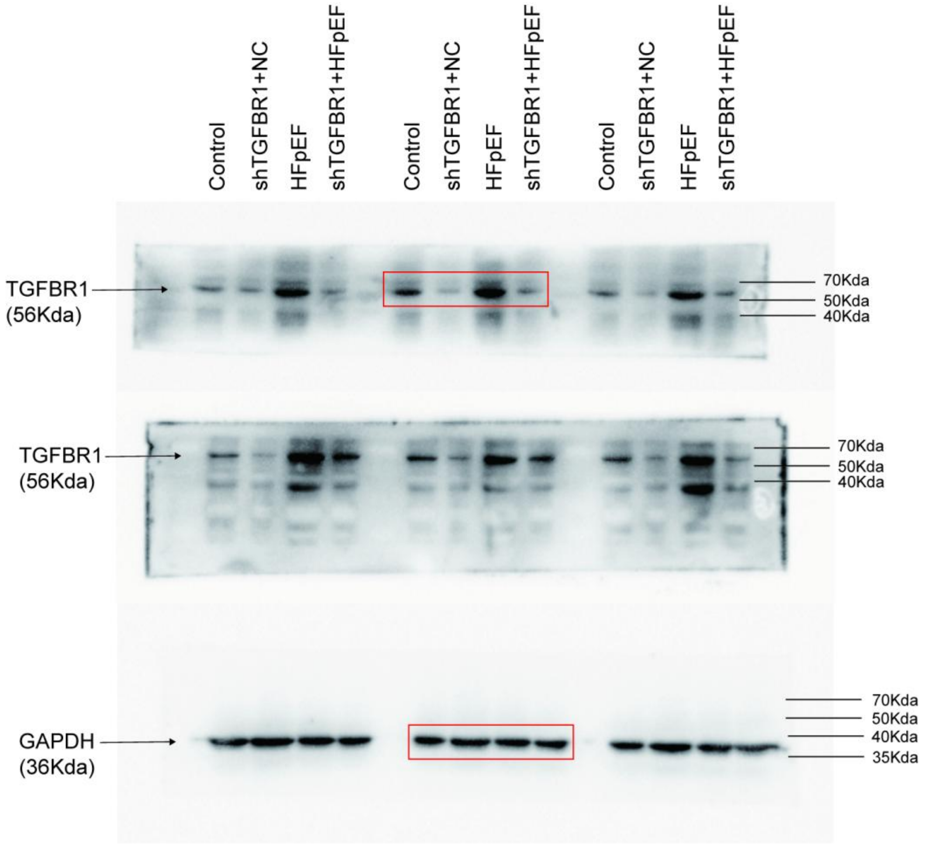

Figure 3K Original Images for Blots

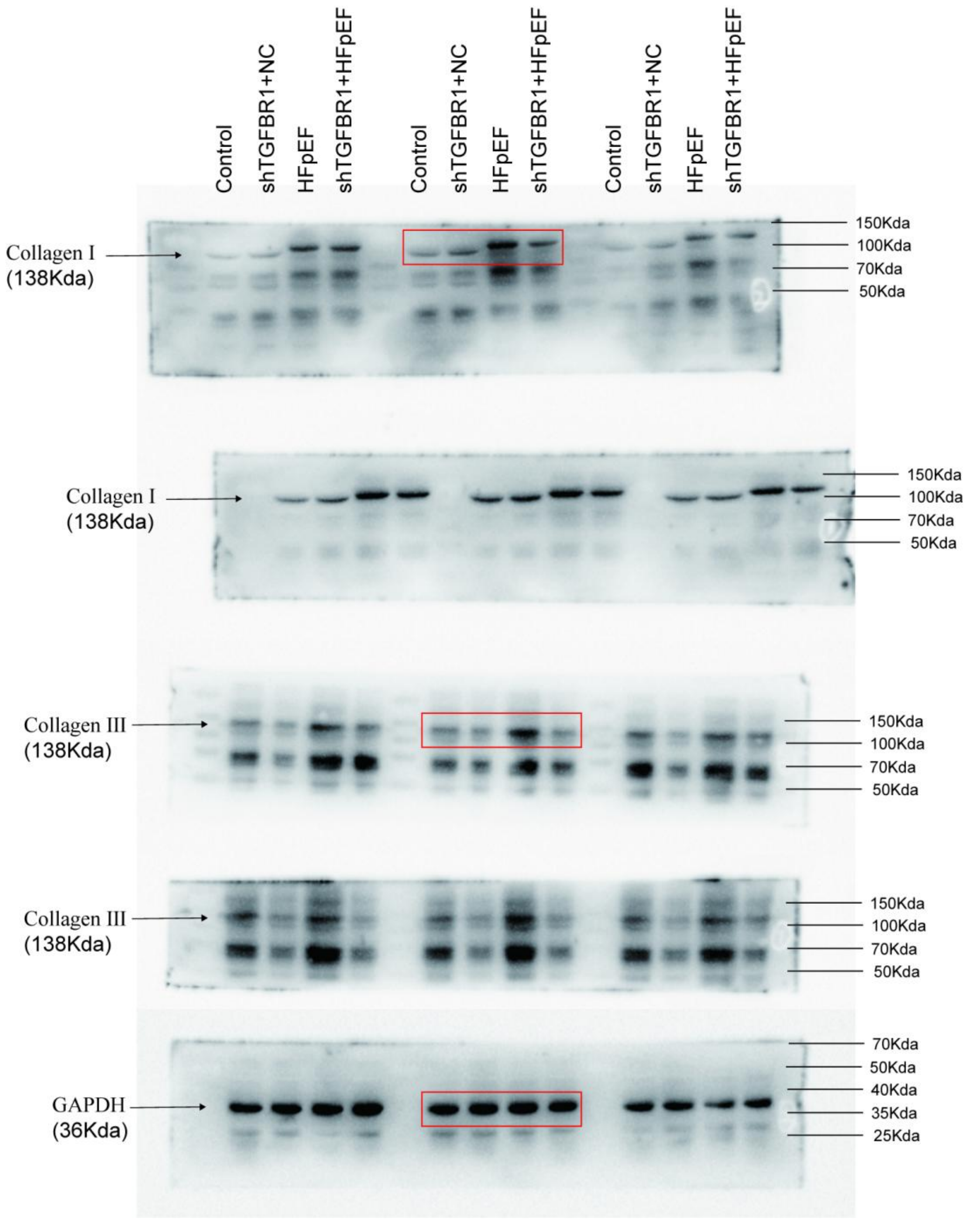

Figure 3K Original Images for Blots

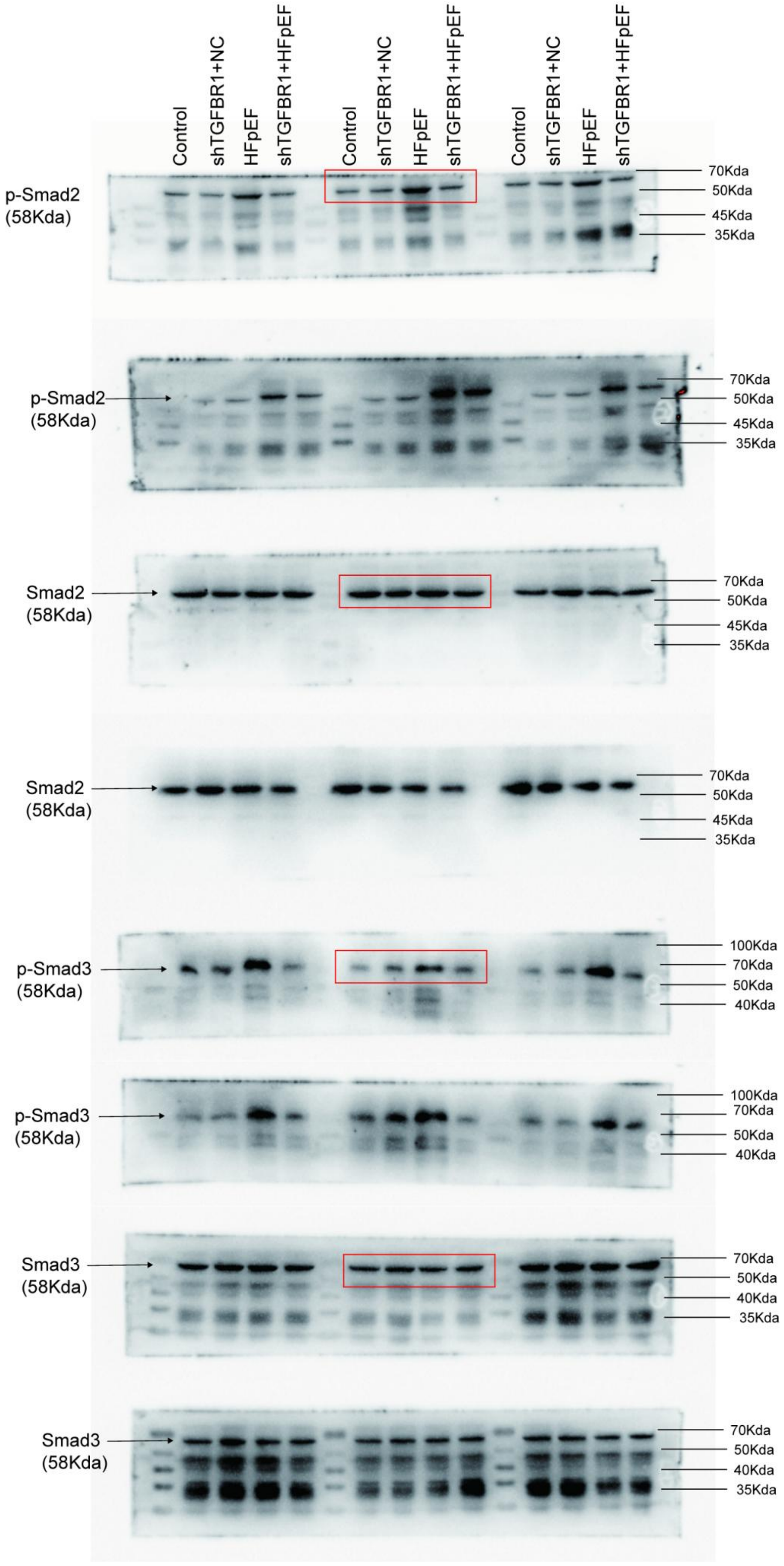

Figure 4A Original Images for Blots

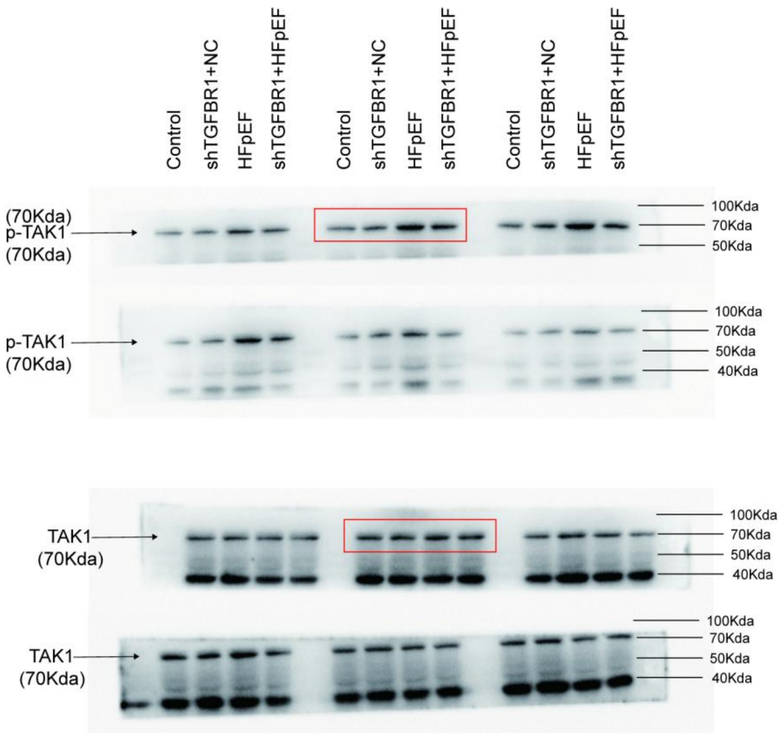

Figure 4E Original Images for Blots

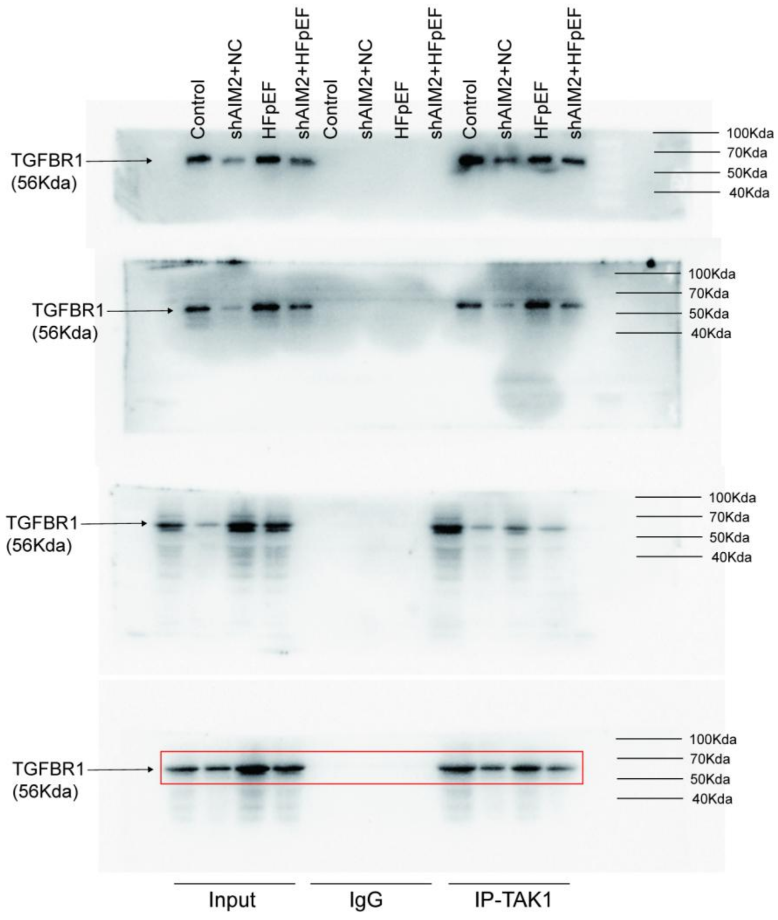

Figure 4E Original Images for Blots

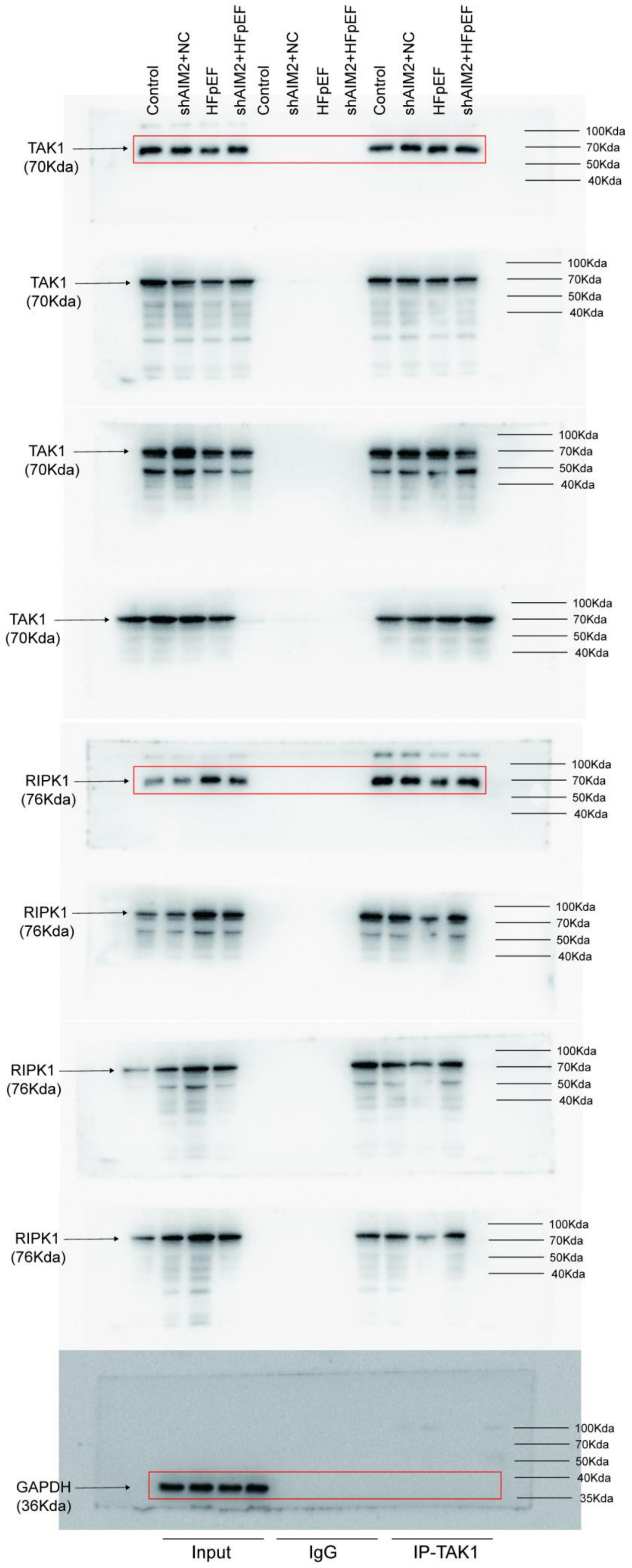

Figure 5C Original Images for Blots

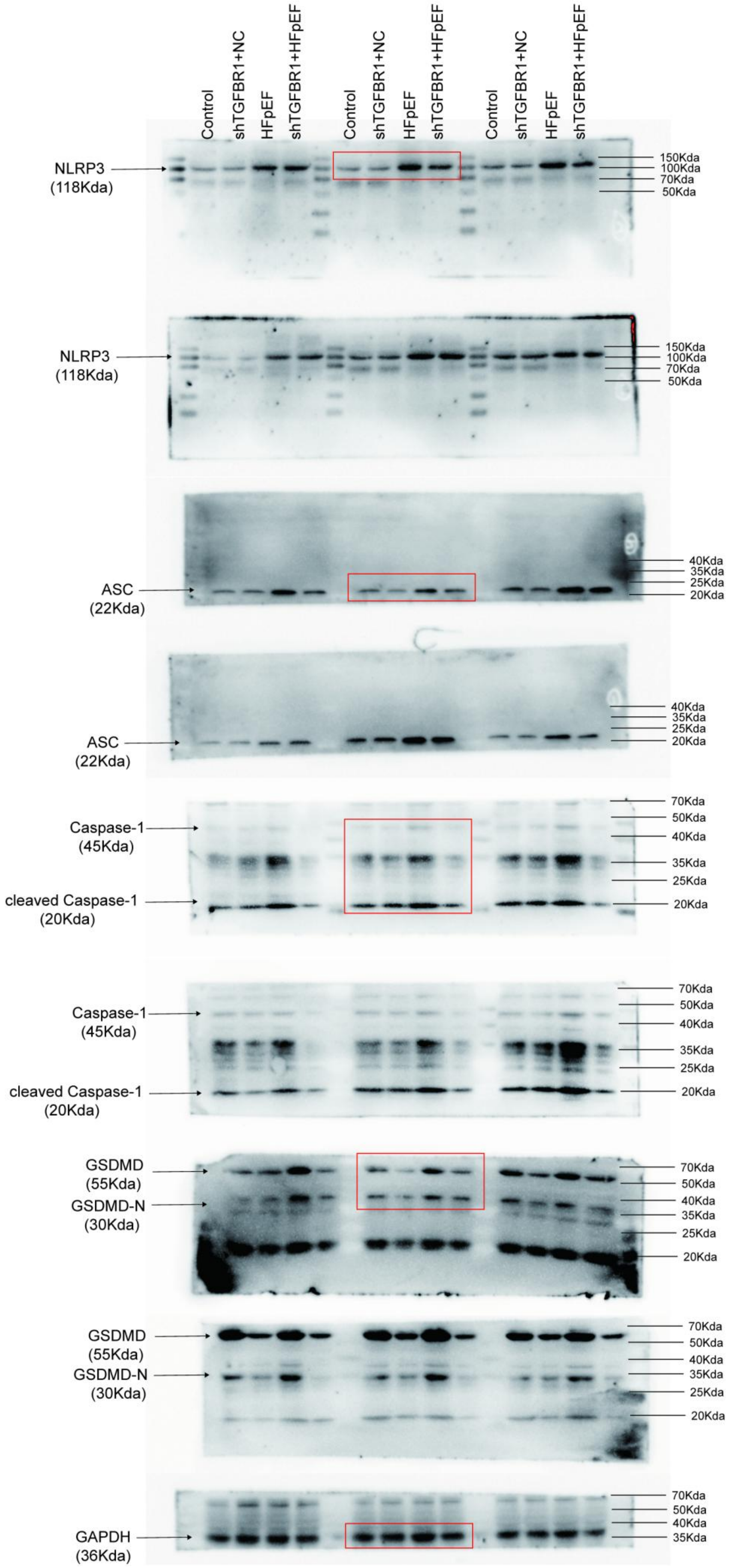

Figure 5D Original Images for Blots

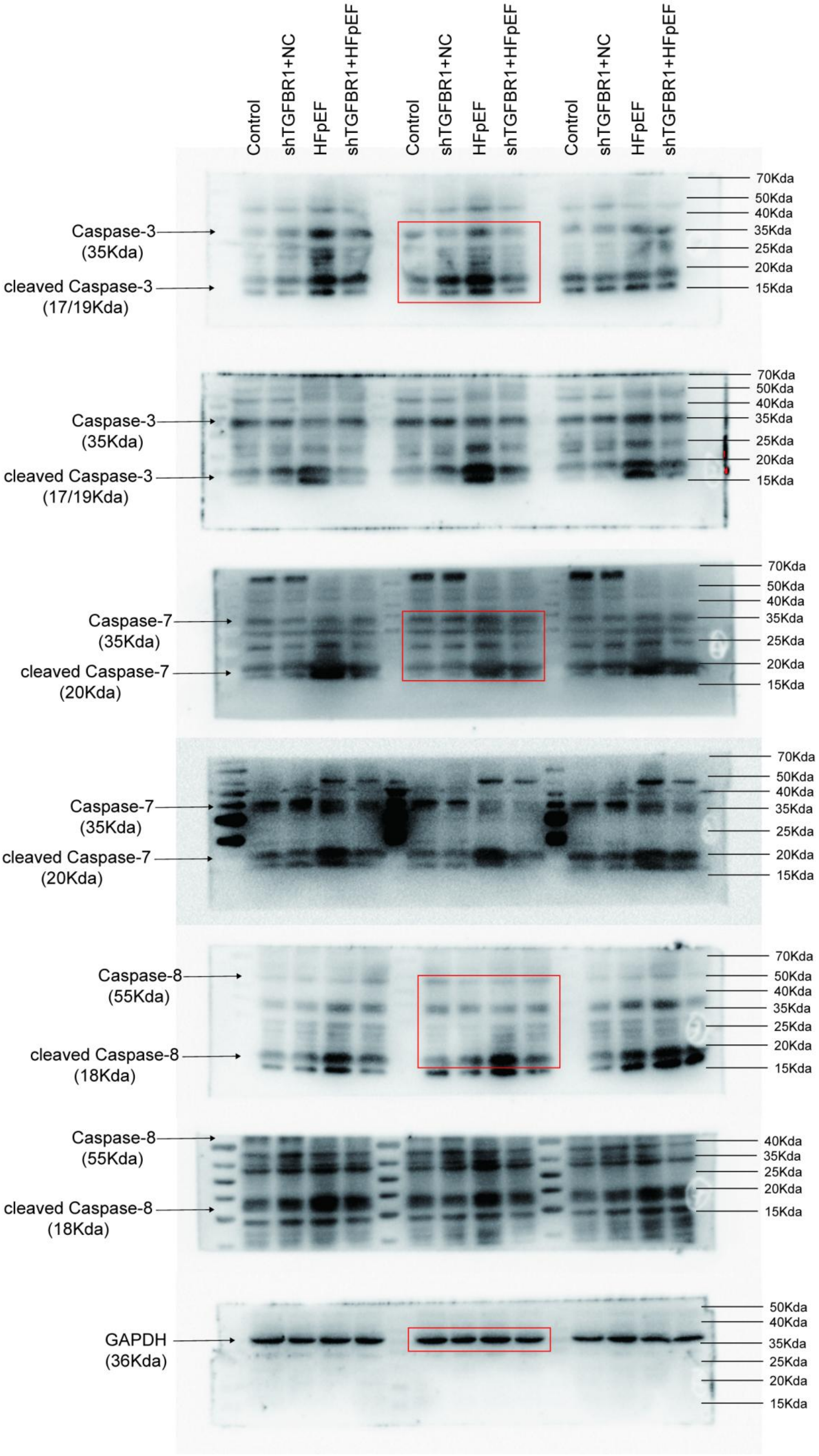

Figure 5E Original Images for Blots

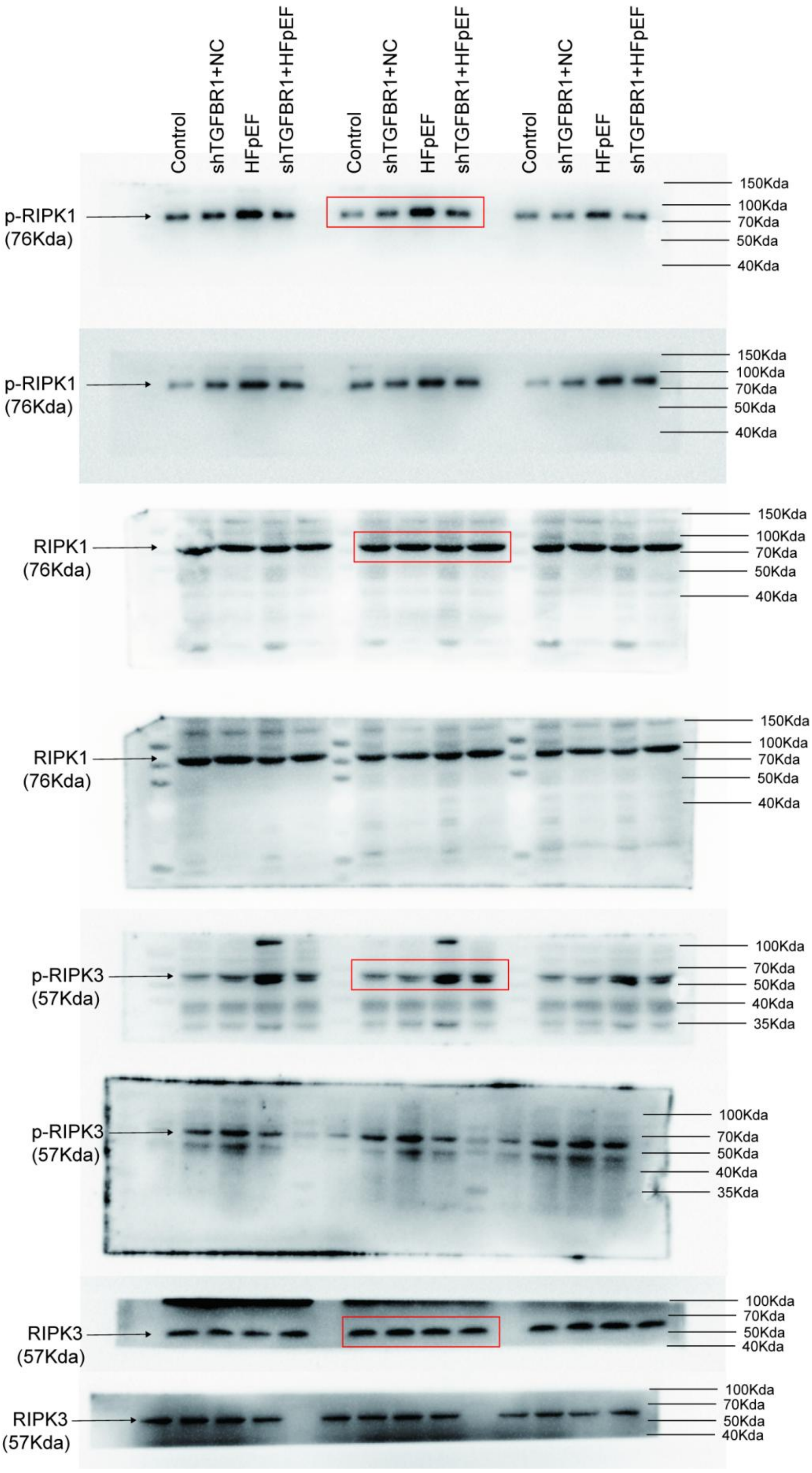

Figure 5E Original Images for Blots

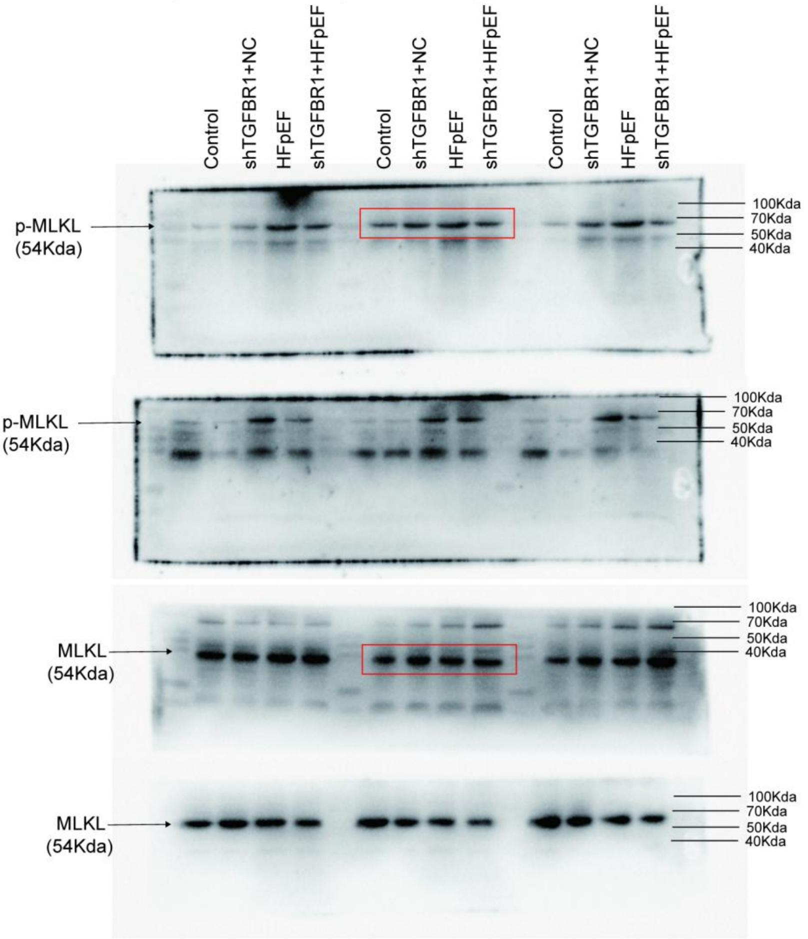

Figure 6A Original Images for Blots

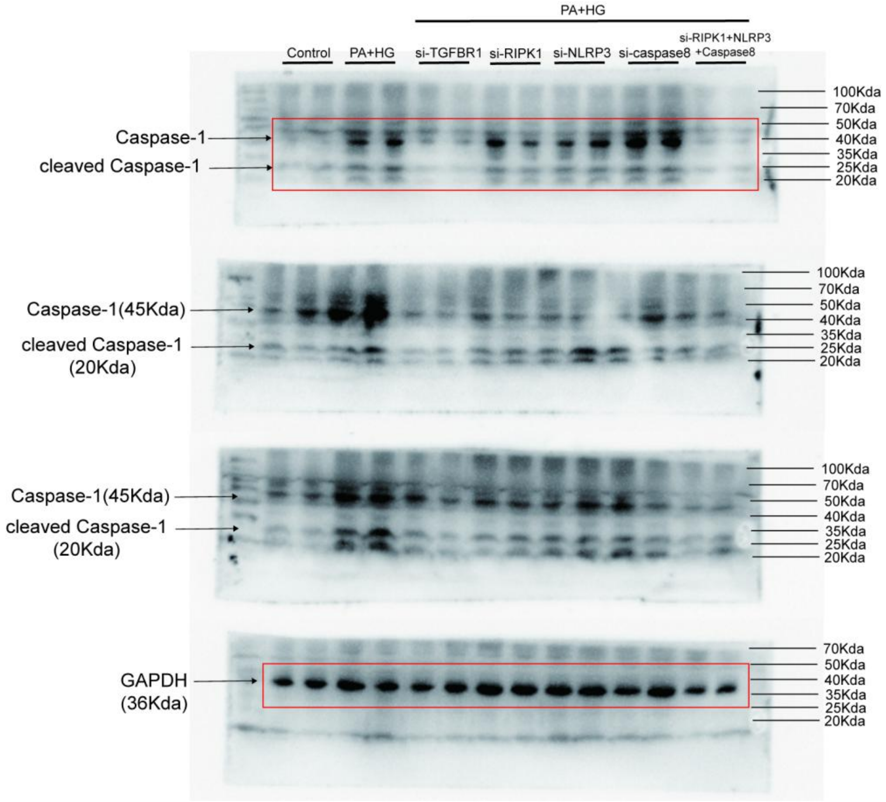

Figure 5E Original Images for Blots

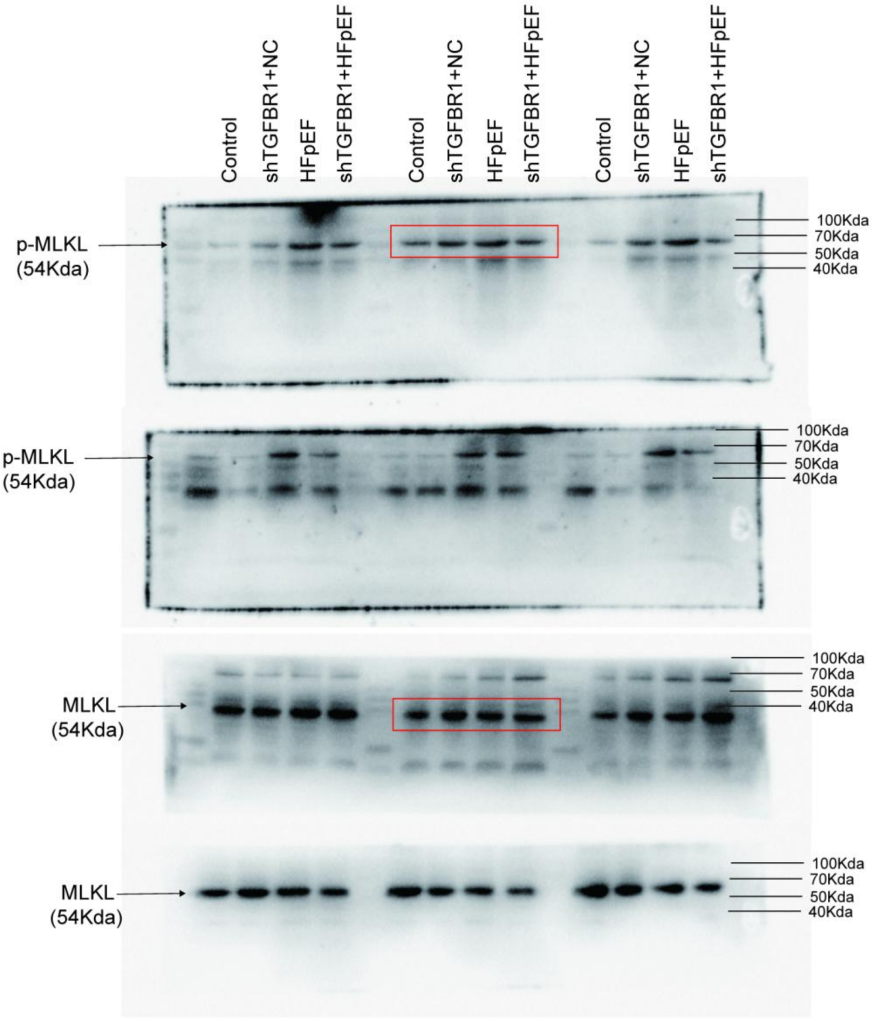

Figure 6A Original Images for Blots

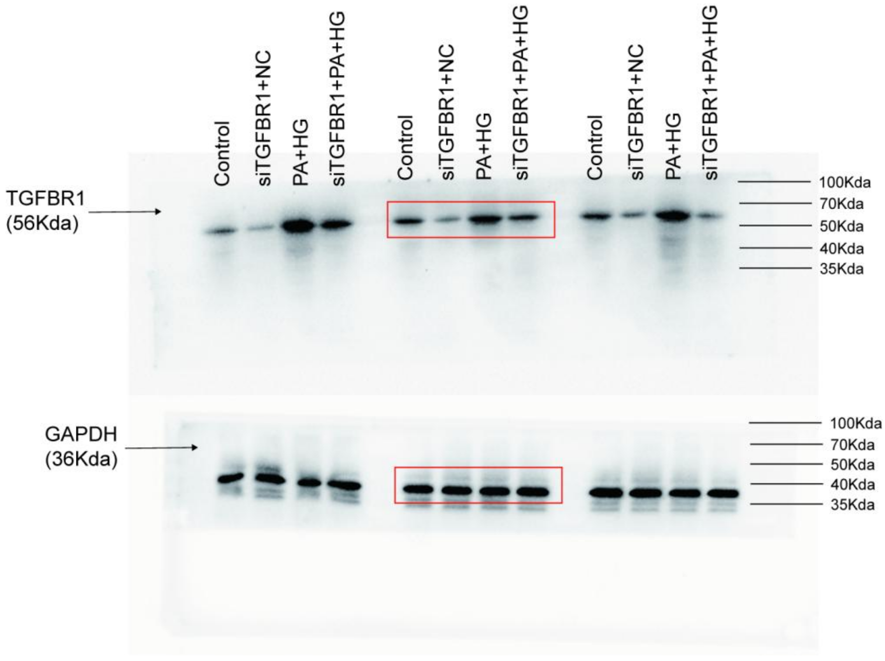

Figure 6A Original Images for Blots

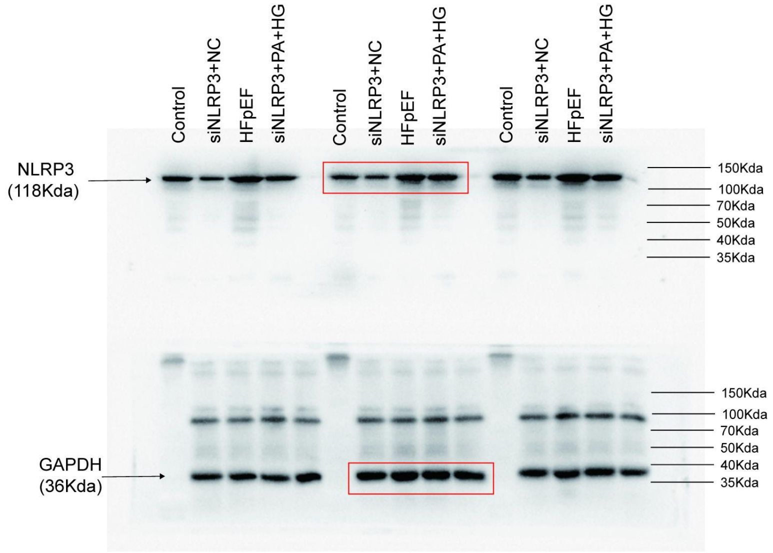

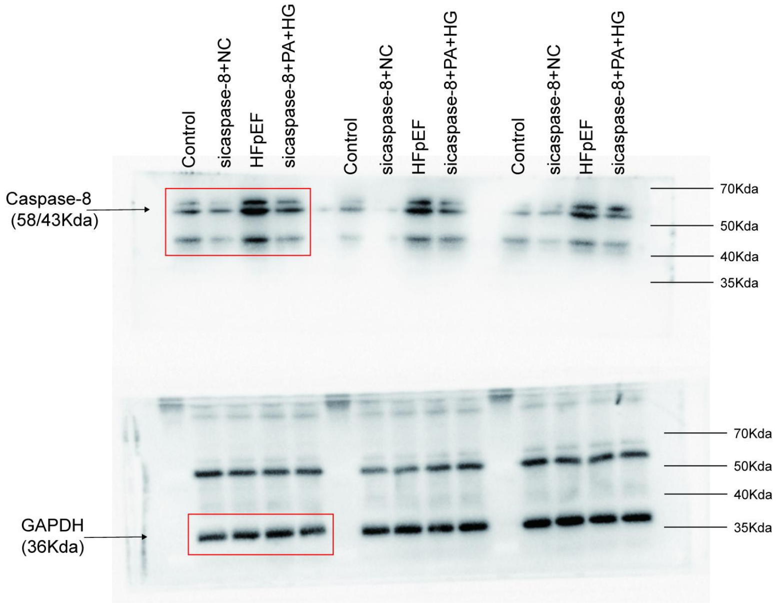

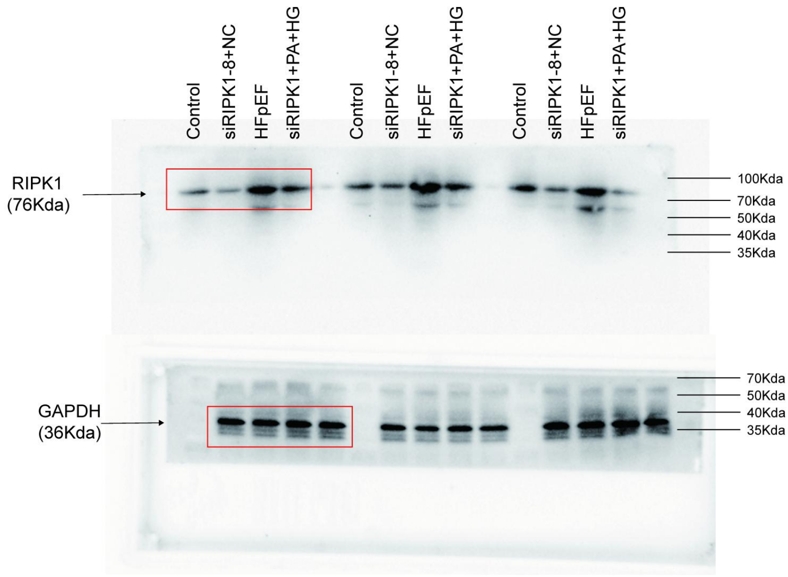

Figure 6C Original Images for Blots

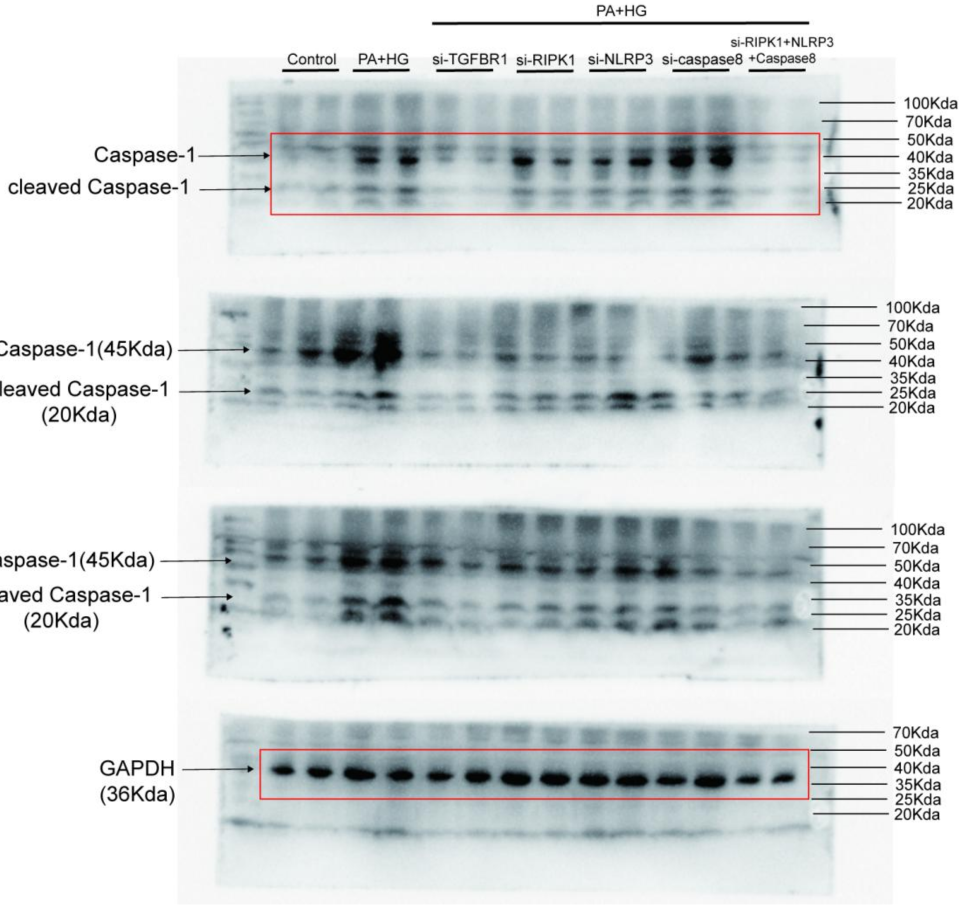

Figure 7B Original Images for Blots

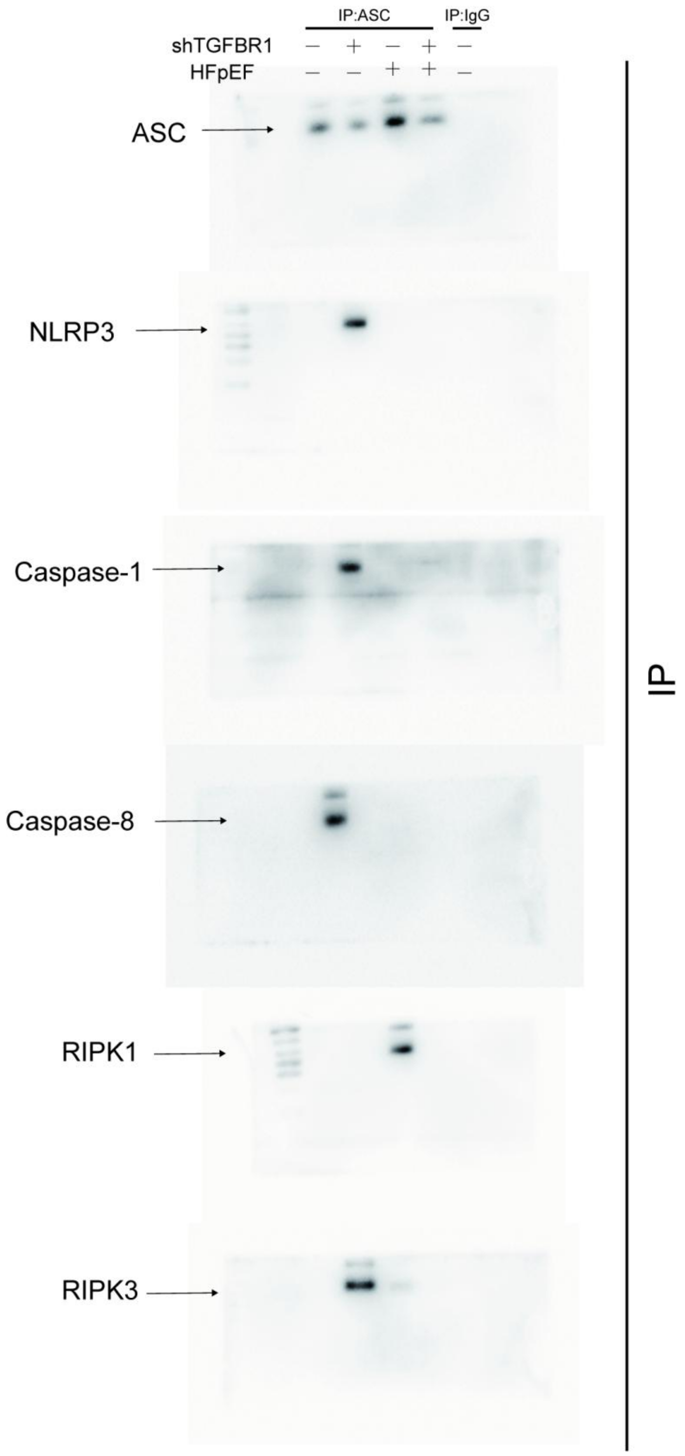

Figure 8A Original Images for Blots

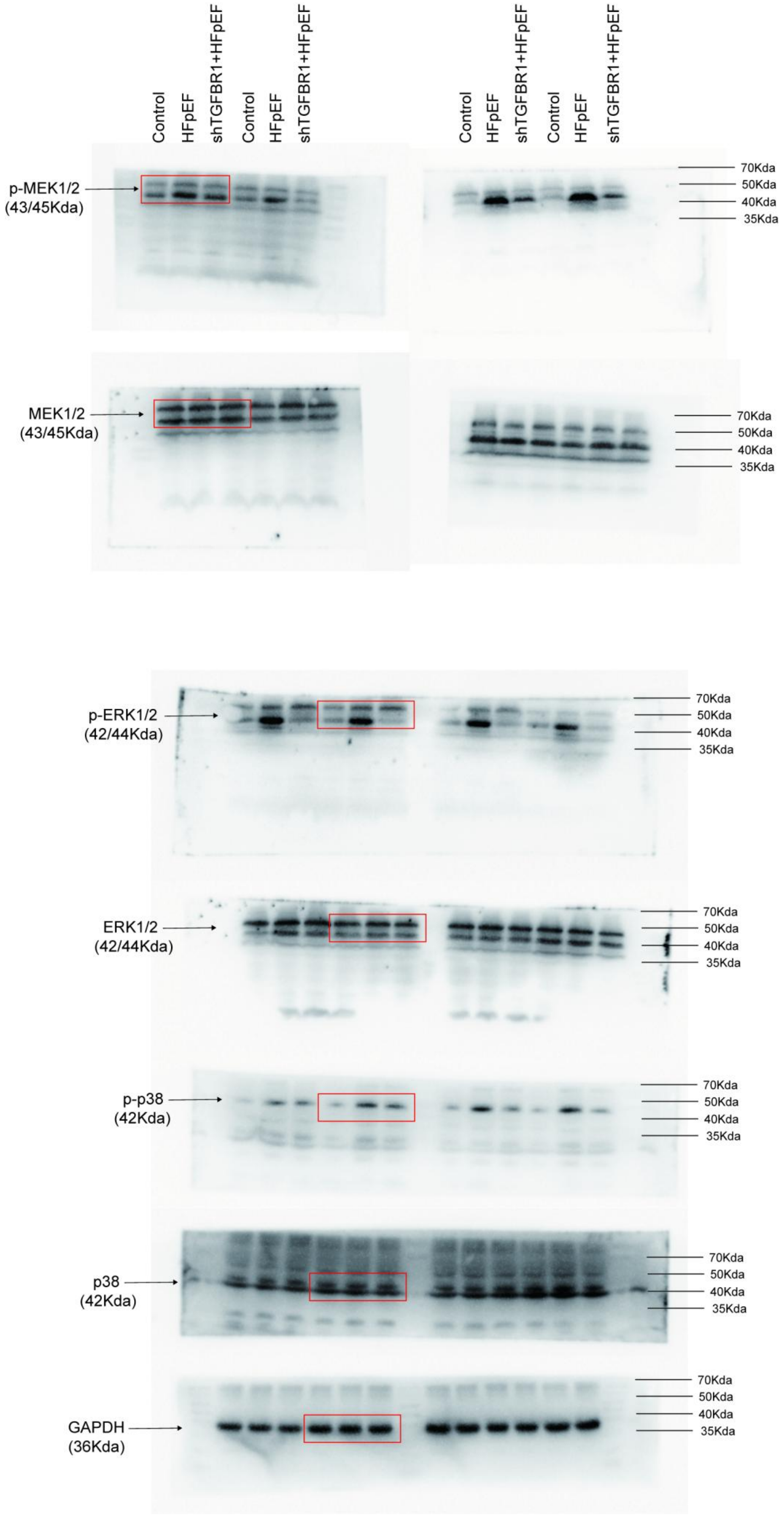

Figure 8A Original Images for Blots

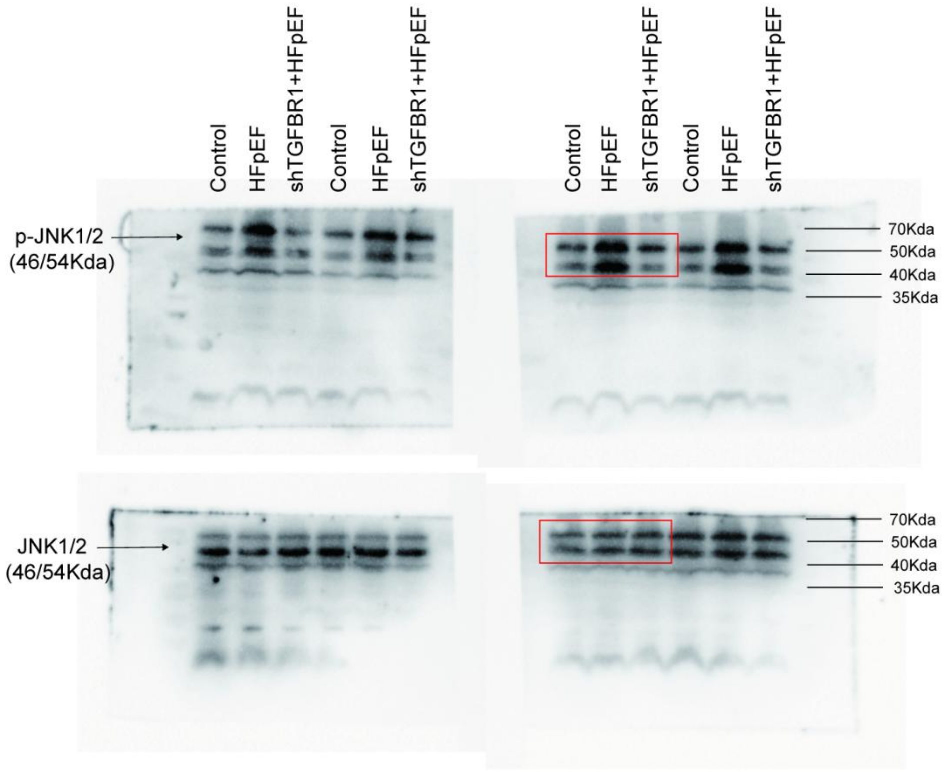

Figure 8C Original Images for Blots

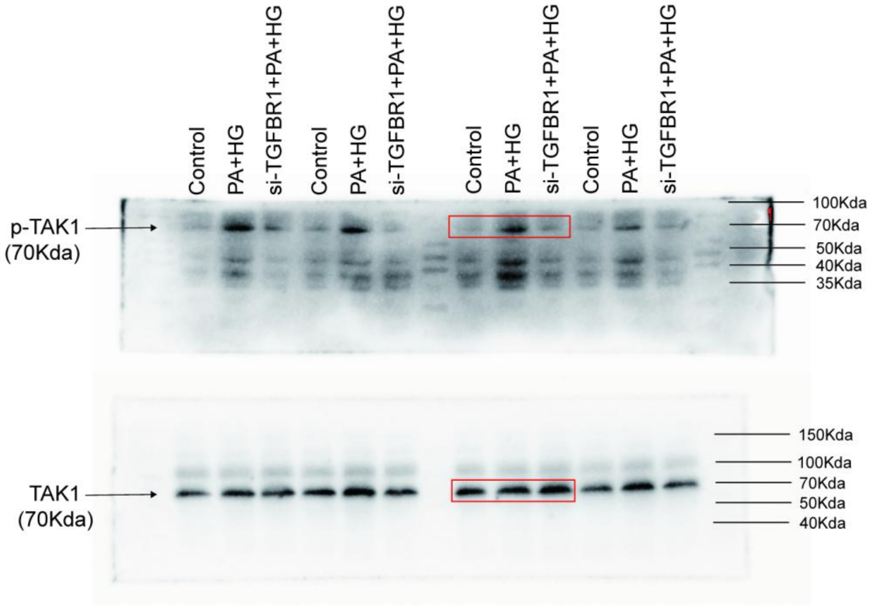

Figure 8C Original Images for Blots

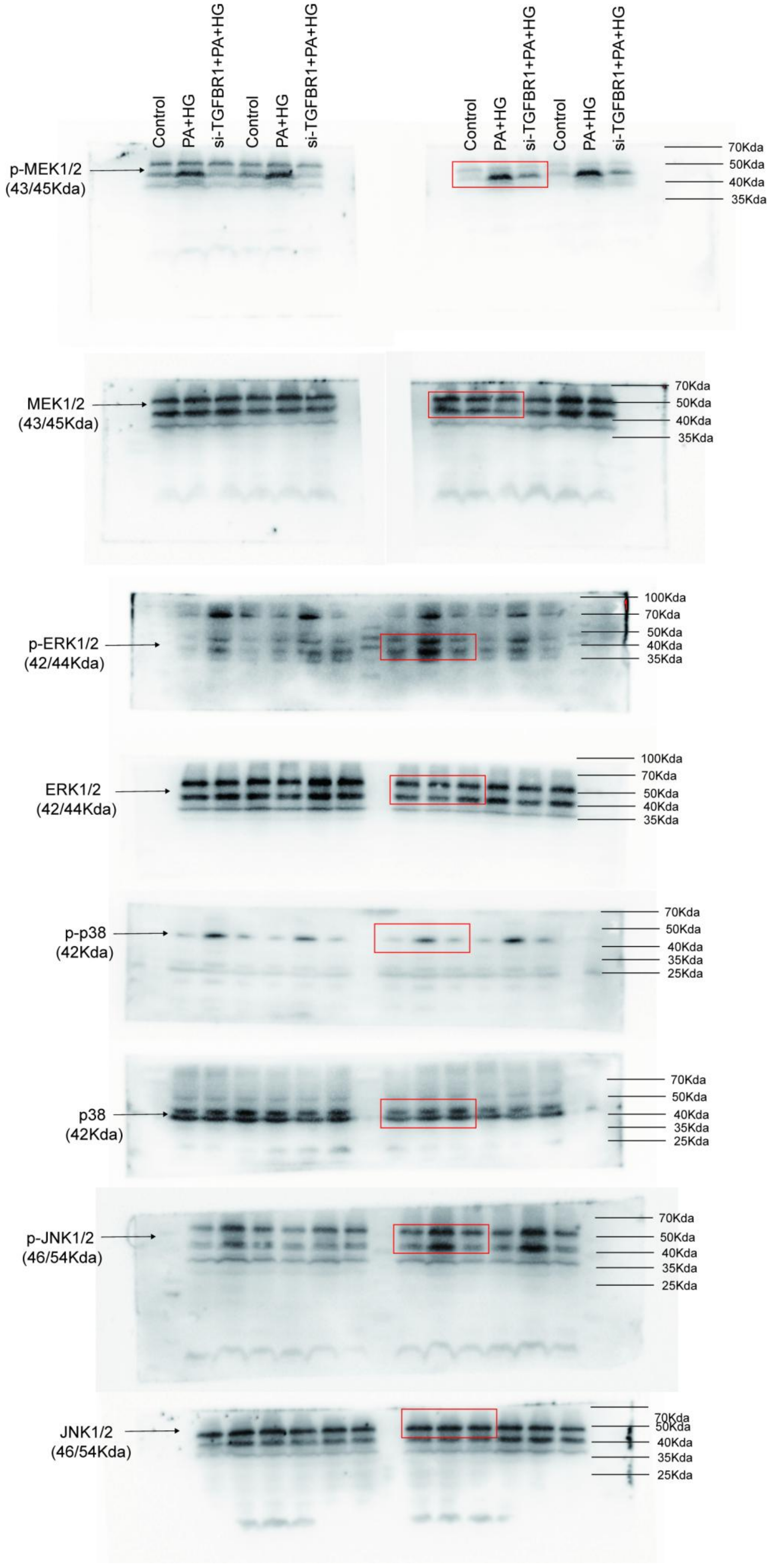

Figure 9A Original Images for Blots

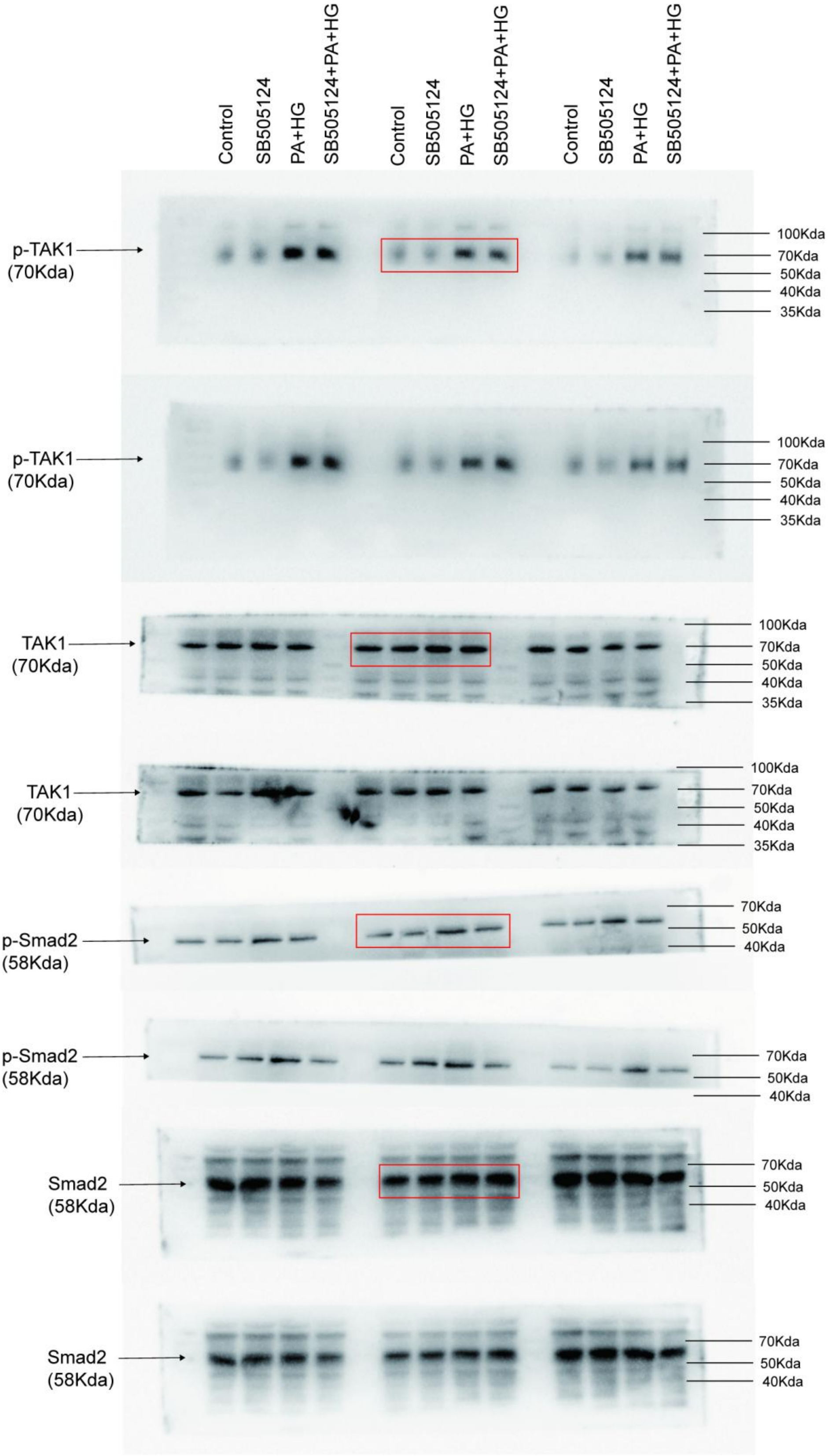

Figure 9A Original Images for Blots

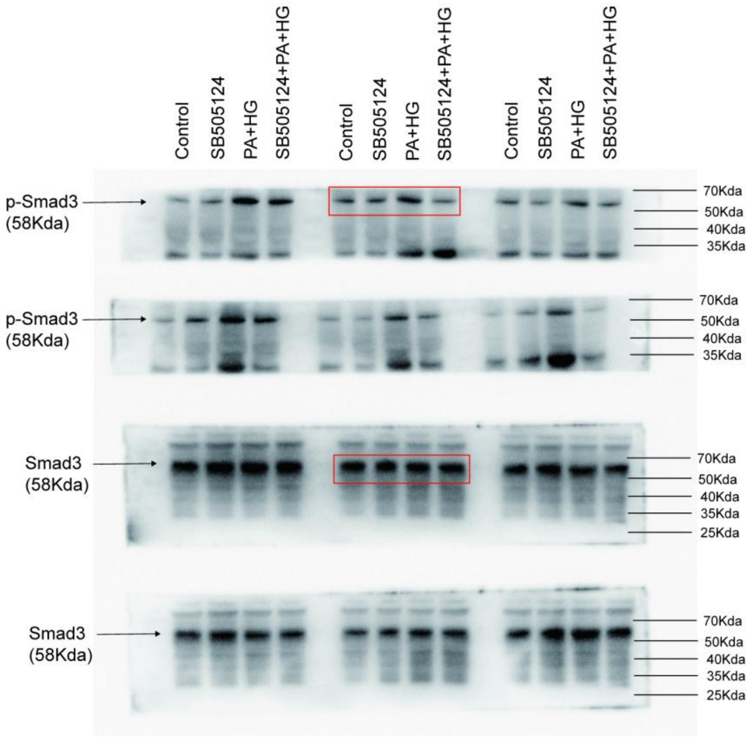

Figure 9C Original Images for Blots

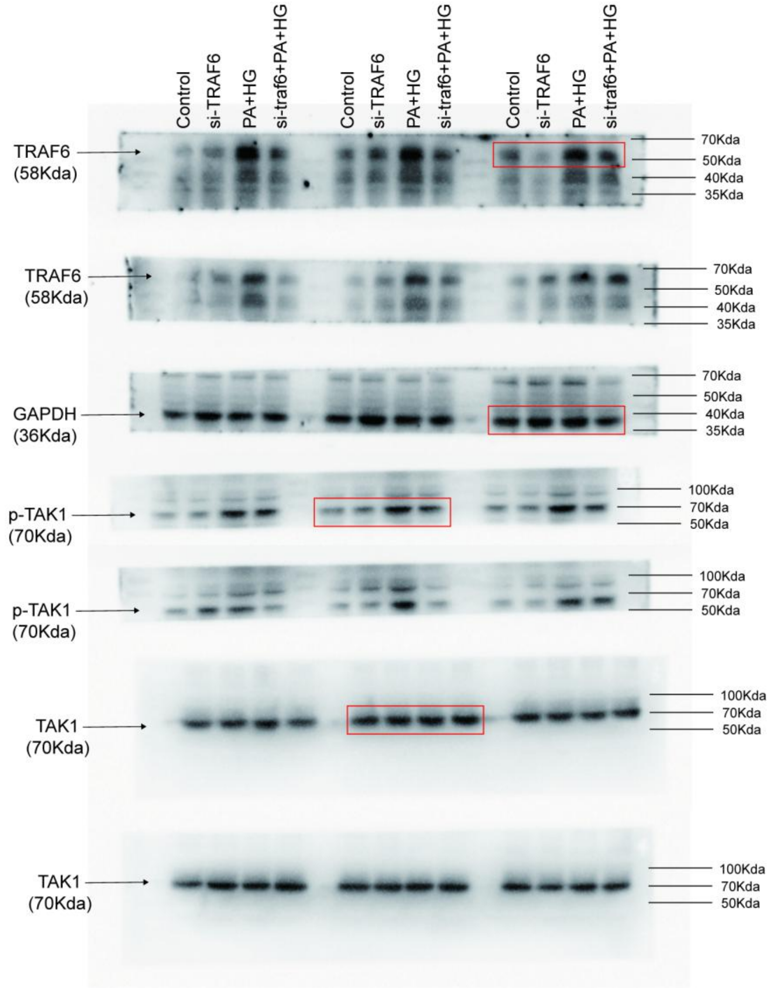

Figure 9E Original Images for Blots

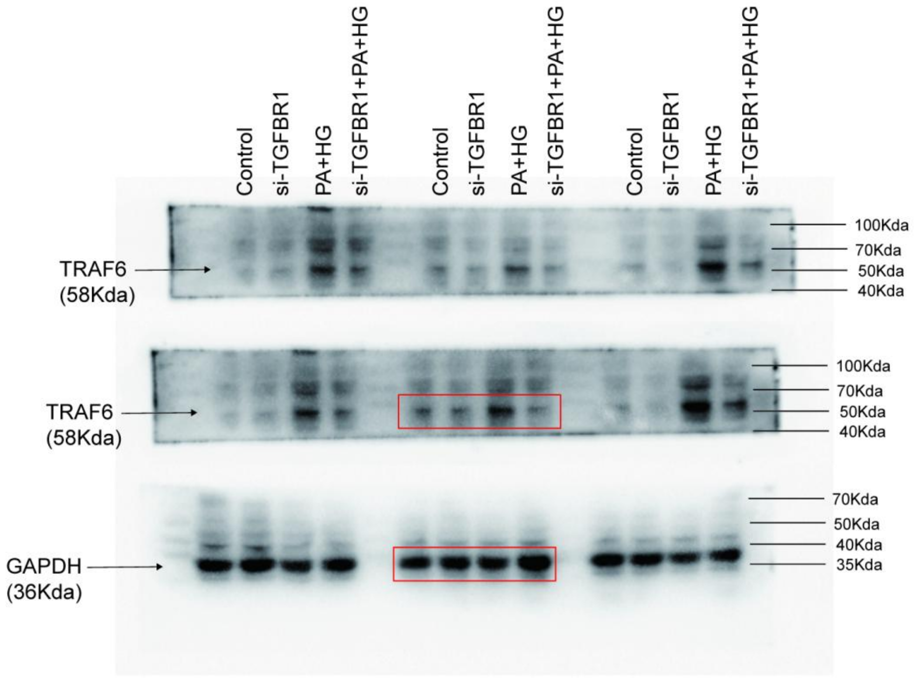

Figure 9I Original Images for Blots

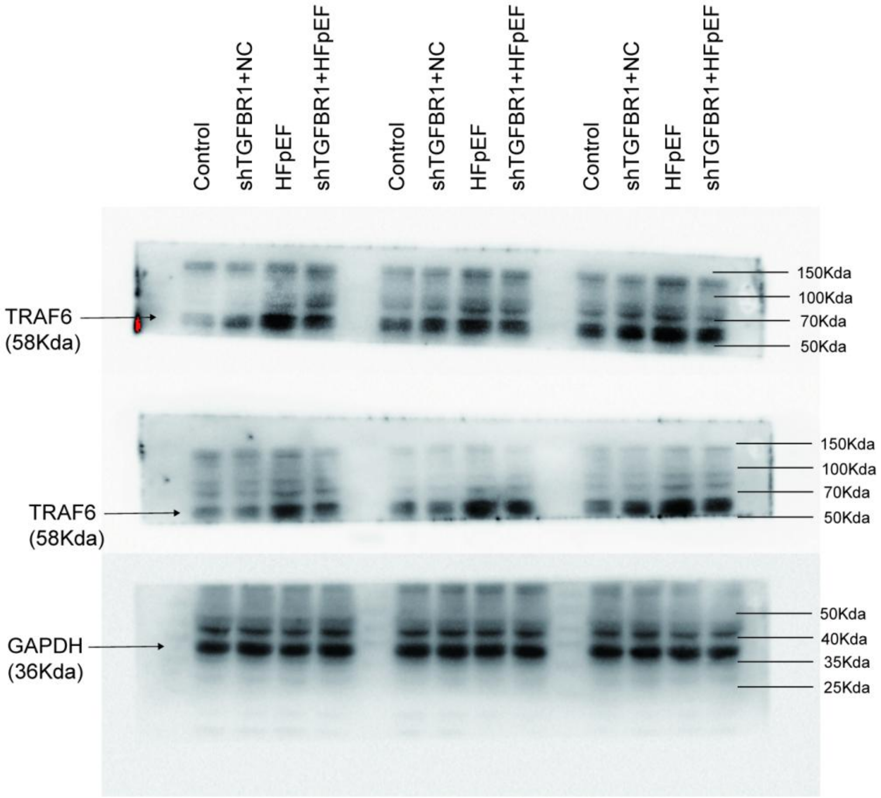

Supplement: S1 File — (PDF) [file pone.0328981.s001.pdf]
