# Supplementary figures and images for: TGFBR1 gene silencing attenuates cardiomyopathy in the HFpEF mouse model
Source: PLoS One. 2025 Aug 29;20(8):e0328981. doi: 10.1371/journal.pone.0328981 (PMC12396675; doi:10.1371/journal.pone.0328981)

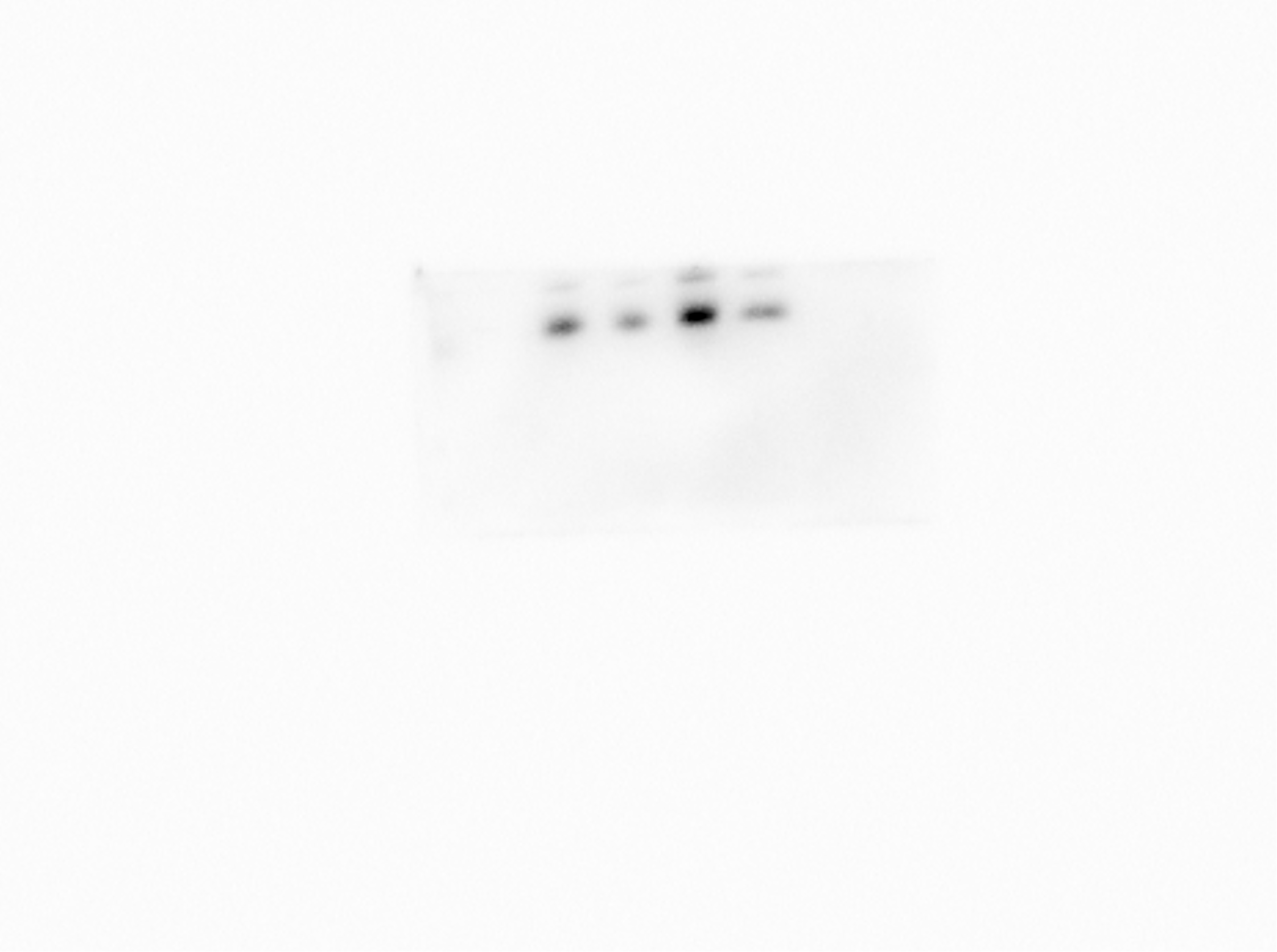

Supplement: S8 File — (ZIP) [file pone.0328981.s008.zip › ASC (Anti-ASC).tif]

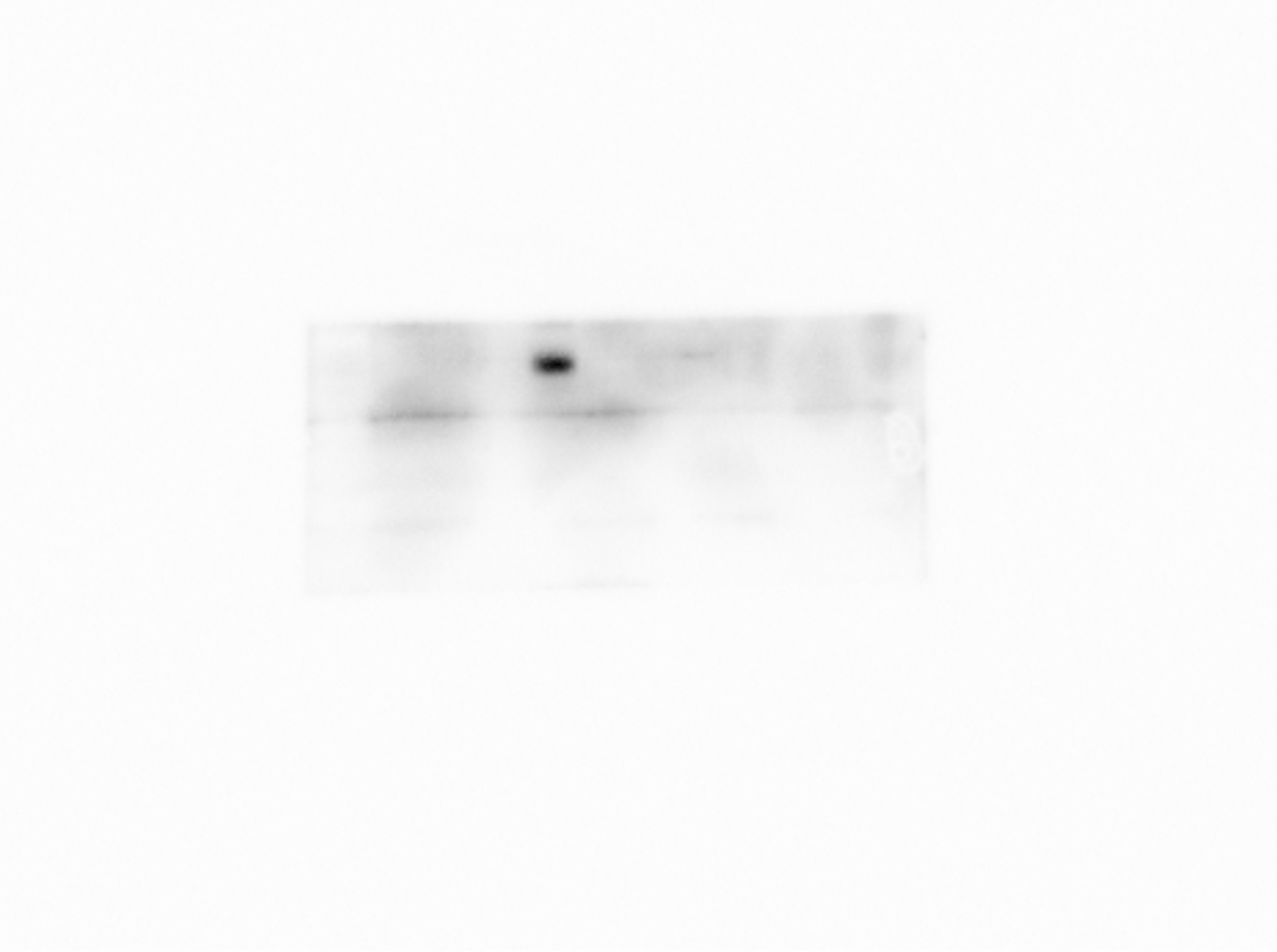

Supplement: S8 File — (ZIP) [file pone.0328981.s008.zip › Caspase1-1 (Anti-ASC).tif]

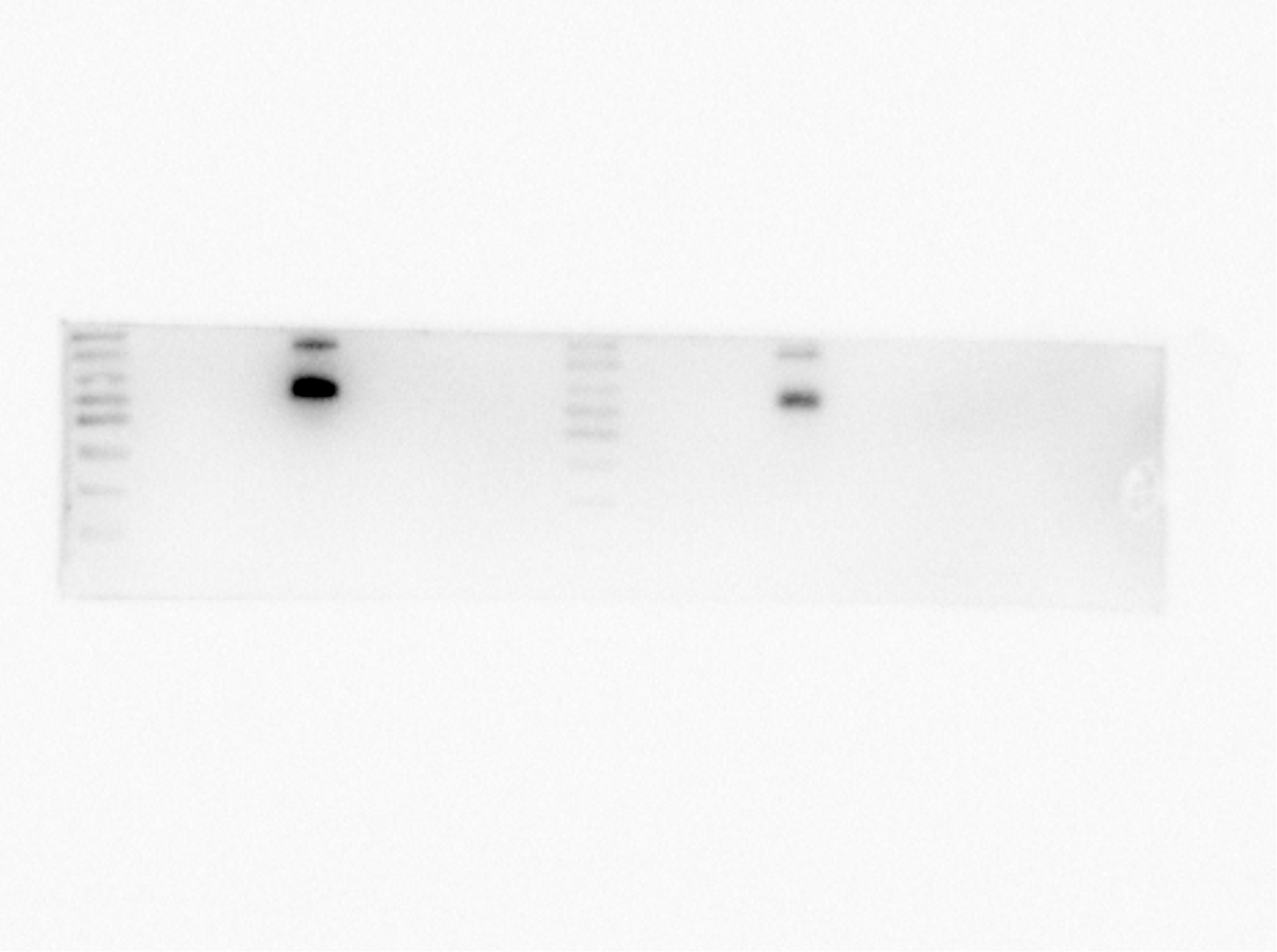

Supplement: S8 File — (ZIP) [file pone.0328981.s008.zip › Caspase1-2 (Anti-ASC).tif]

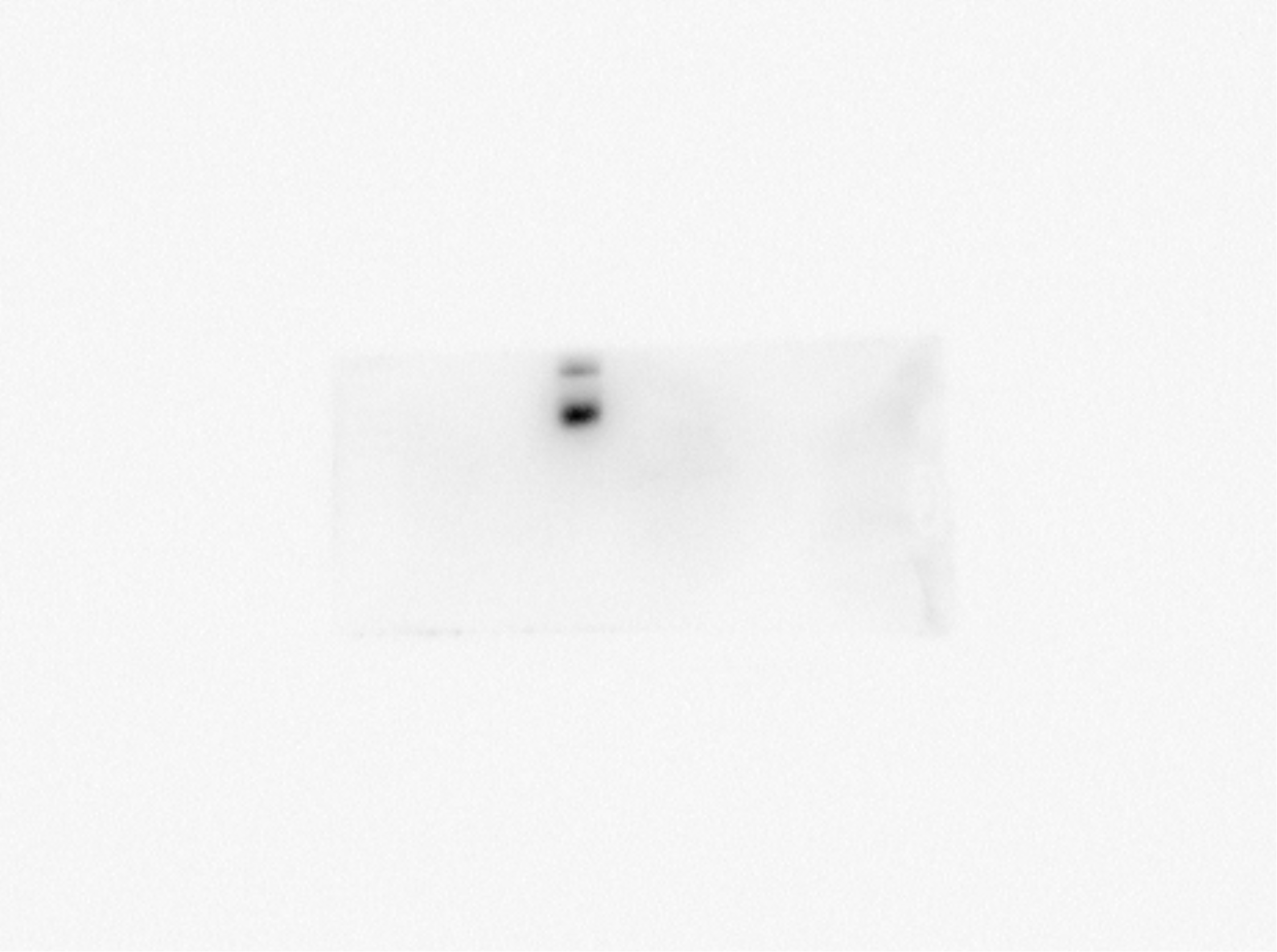

Supplement: S8 File — (ZIP) [file pone.0328981.s008.zip › Caspase8-1 (Anti-ASC).tif]

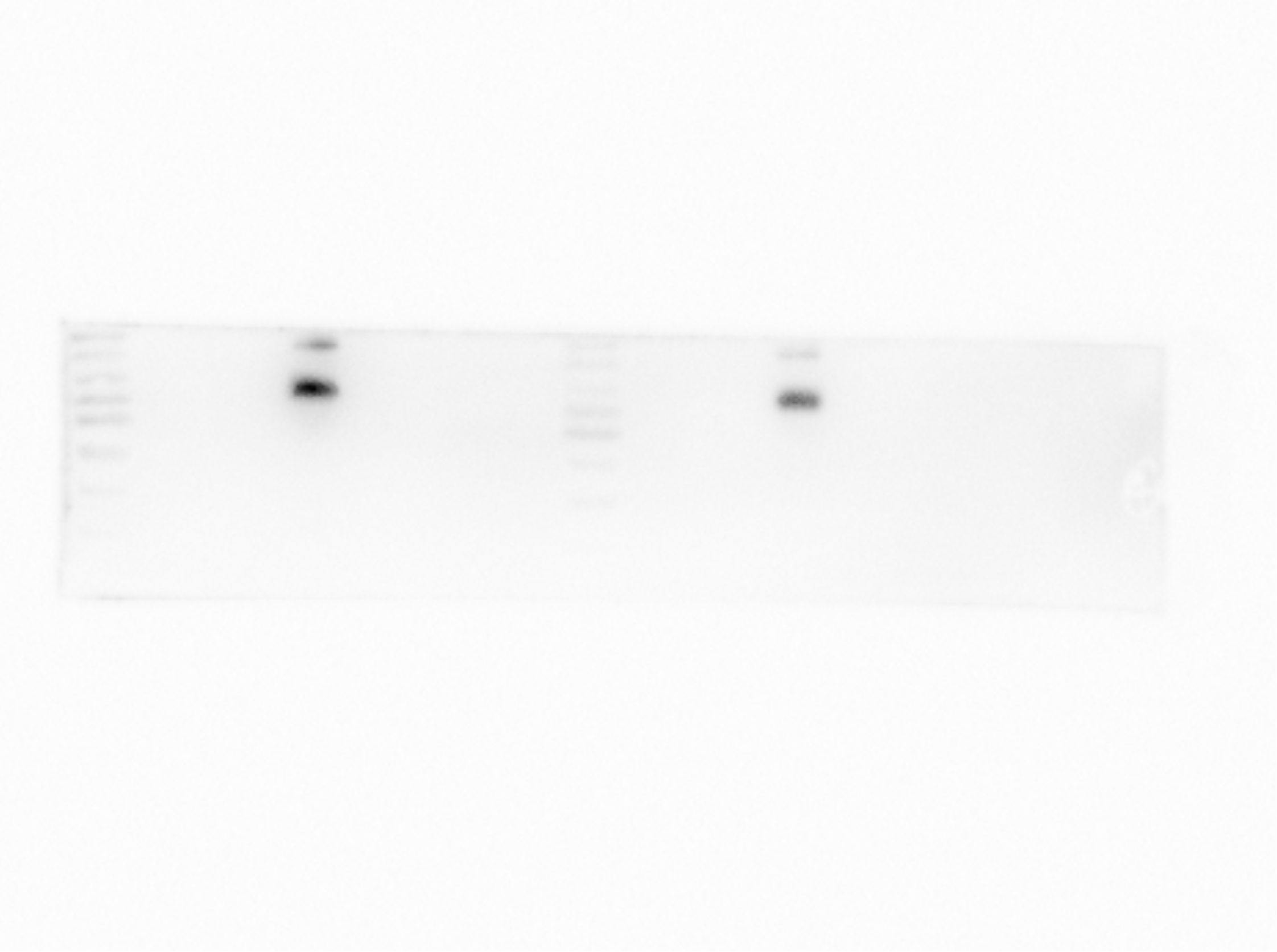

Supplement: S8 File — (ZIP) [file pone.0328981.s008.zip › Caspase8-2 (Anti-ASC).tif]

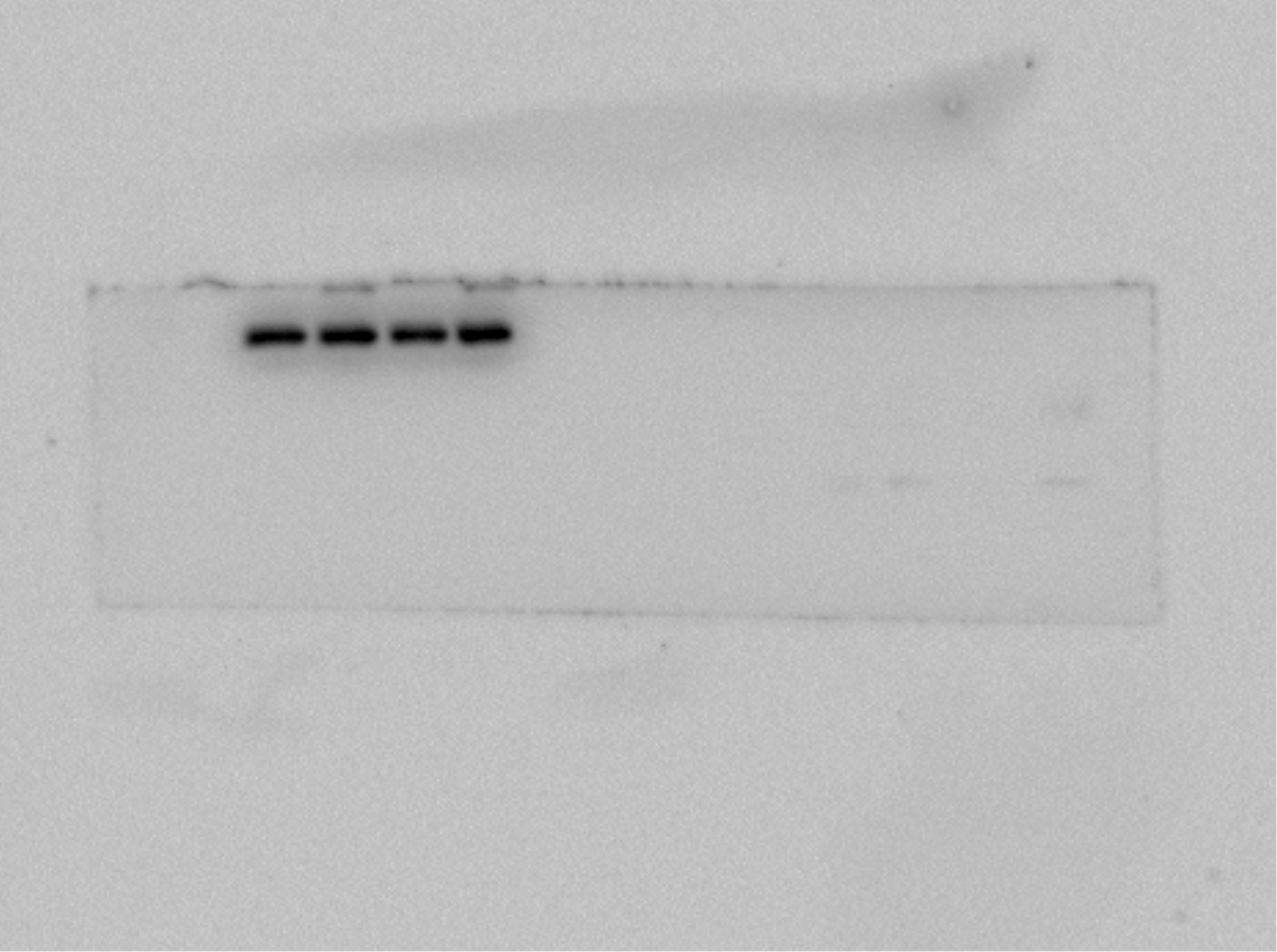

Supplement: S8 File — (ZIP) [file pone.0328981.s008.zip › GAPDH (Anti-ASC).tif]

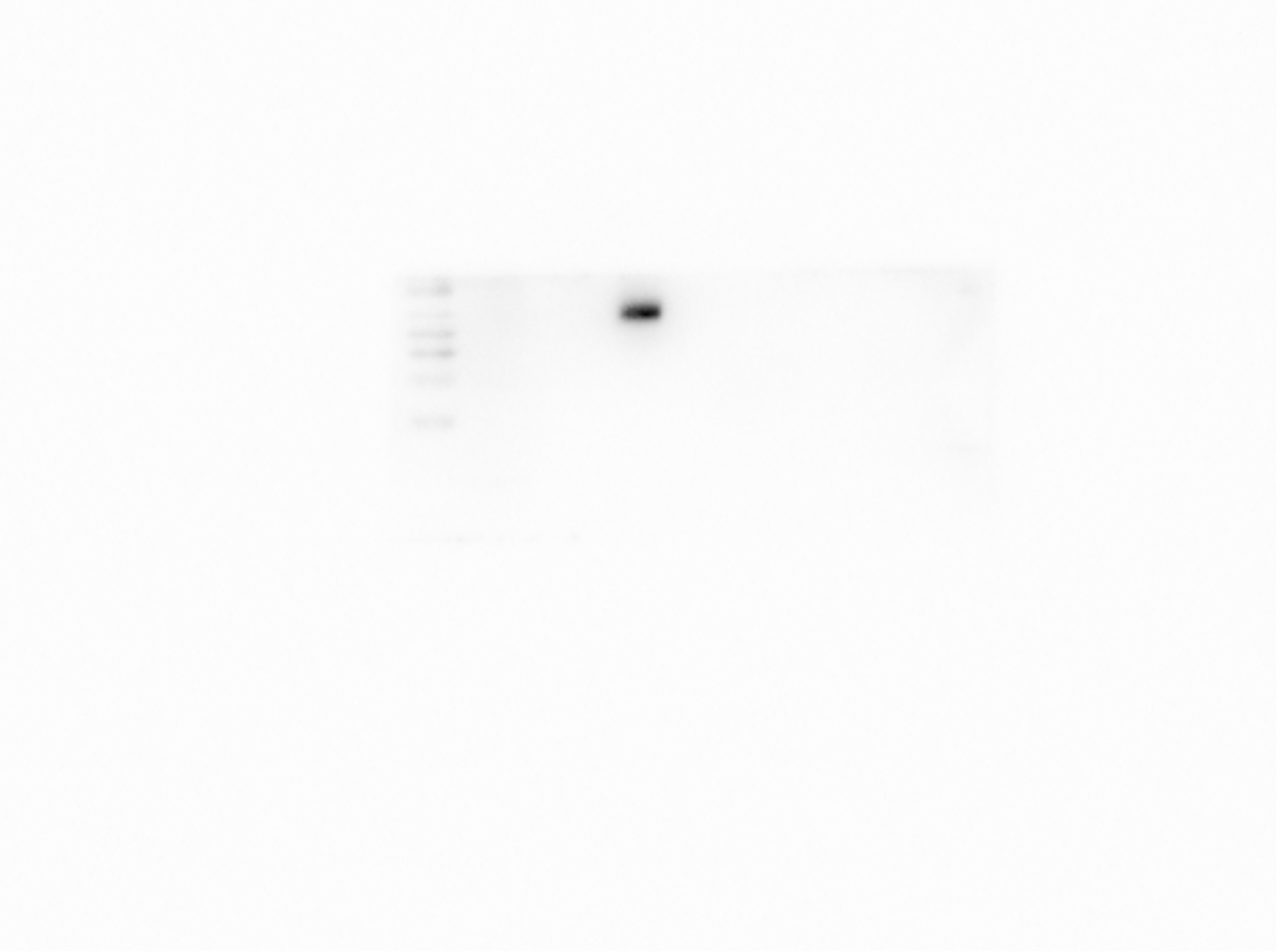

Supplement: S8 File — (ZIP) [file pone.0328981.s008.zip › NLRP3 -1 (Anti-ASC).tif]

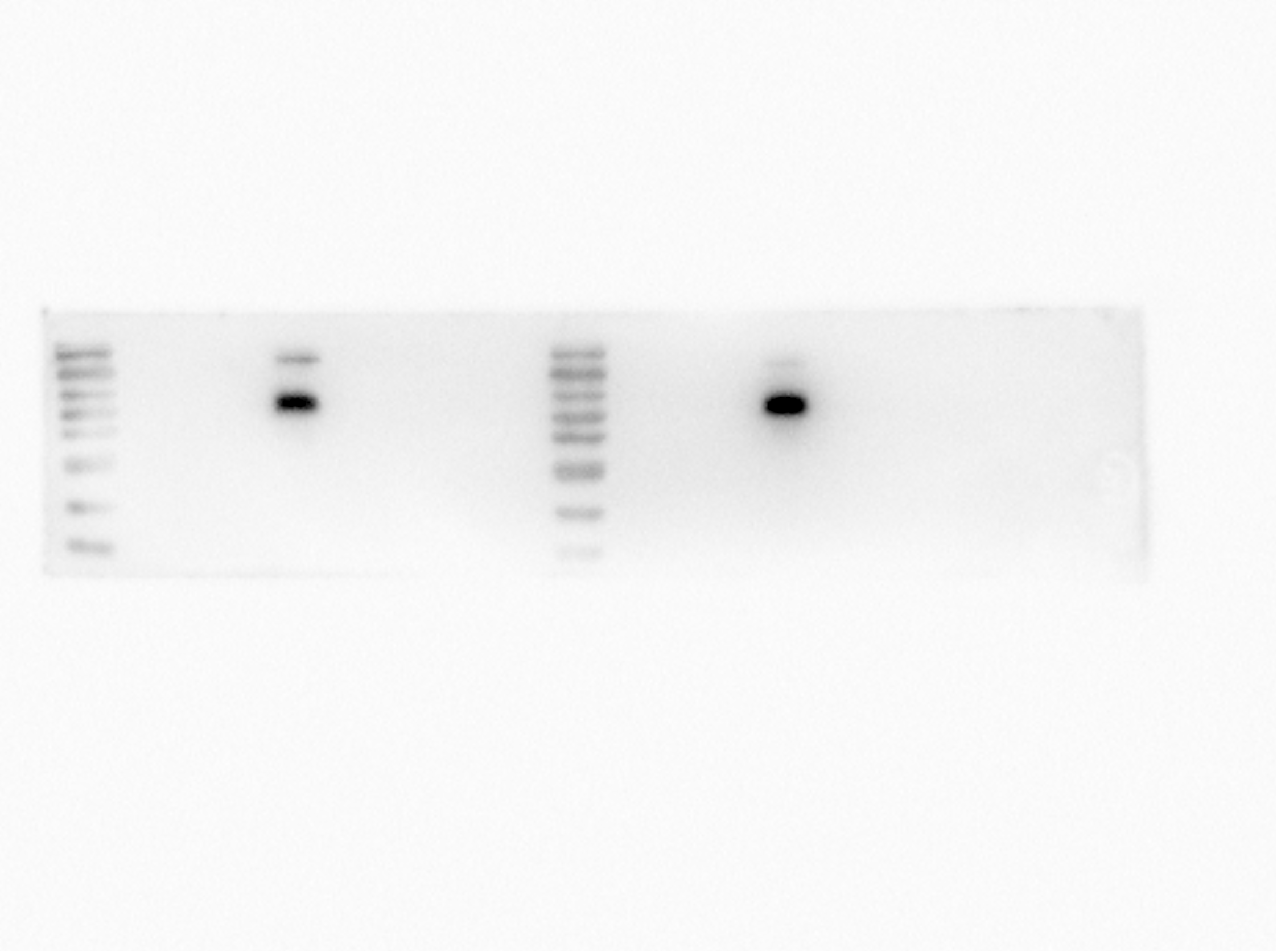

Supplement: S8 File — (ZIP) [file pone.0328981.s008.zip › NLRP3 -2 (Anti-ASC).tif]

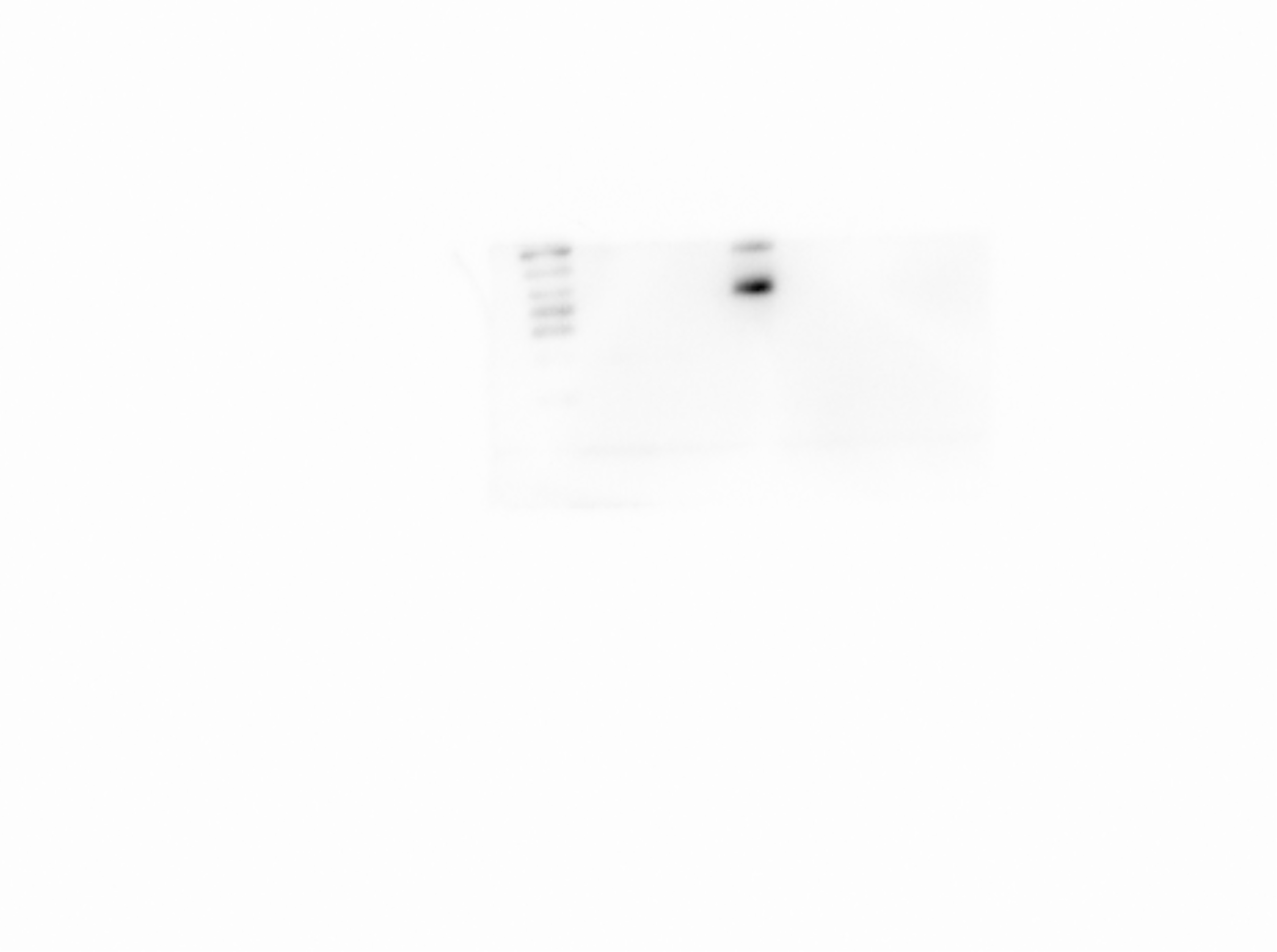

Supplement: S8 File — (ZIP) [file pone.0328981.s008.zip › RIPK1-1 (Anti-ASC).tif]

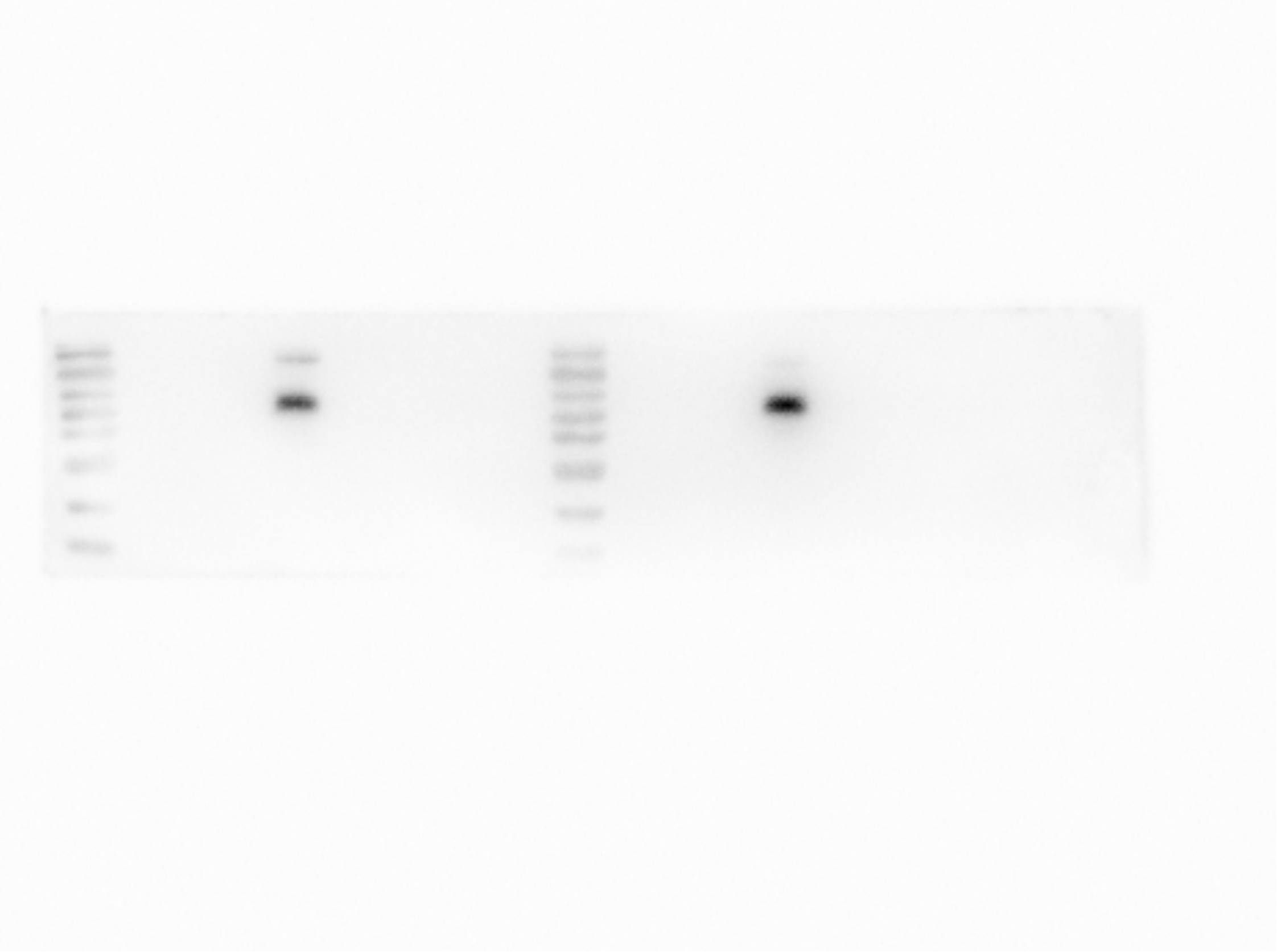

Supplement: S8 File — (ZIP) [file pone.0328981.s008.zip › RIPK1-2 (Anti-ASC).tif]

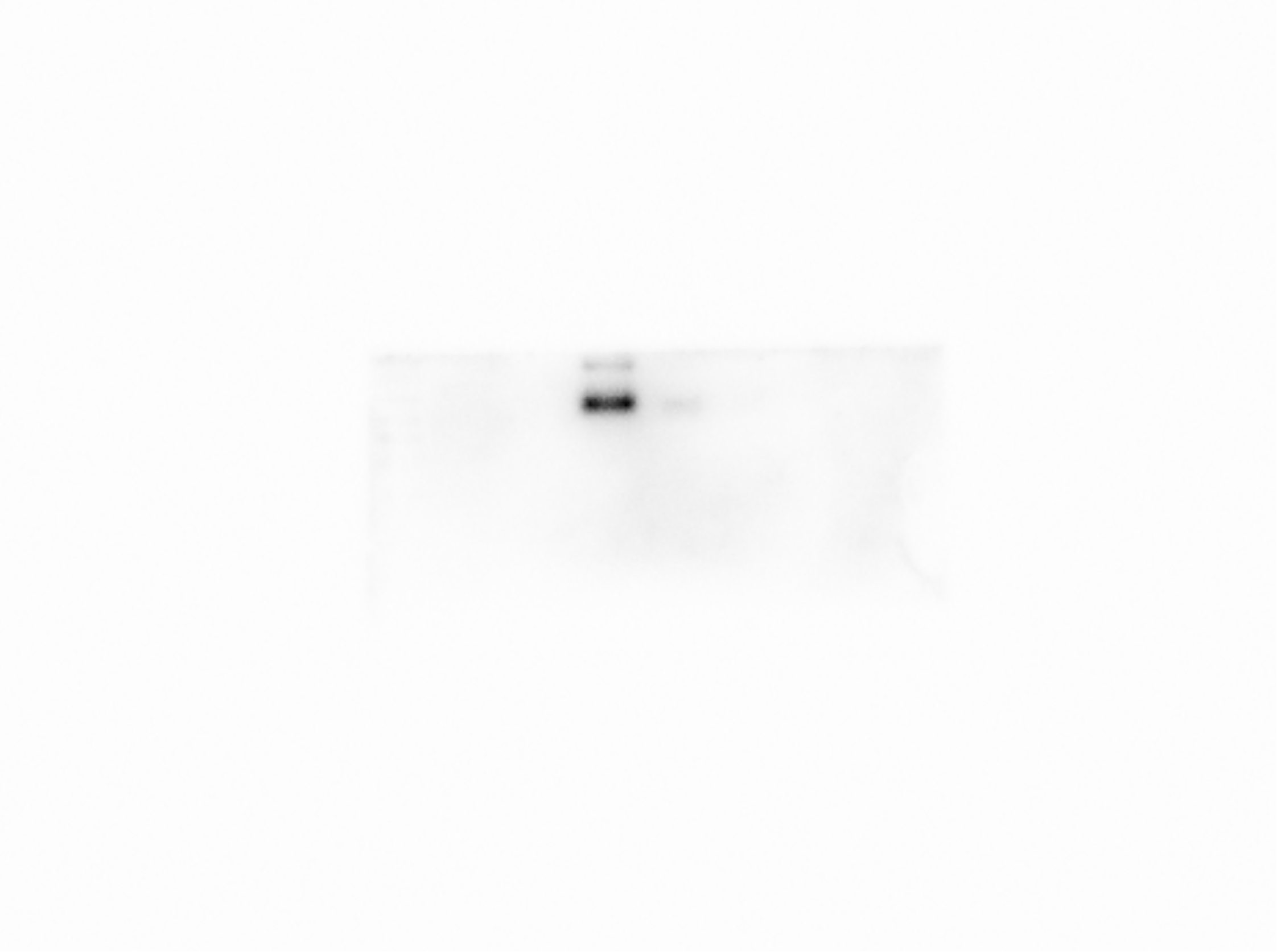

Supplement: S8 File — (ZIP) [file pone.0328981.s008.zip › RIPK3-1 (Anti-ASC).tif]

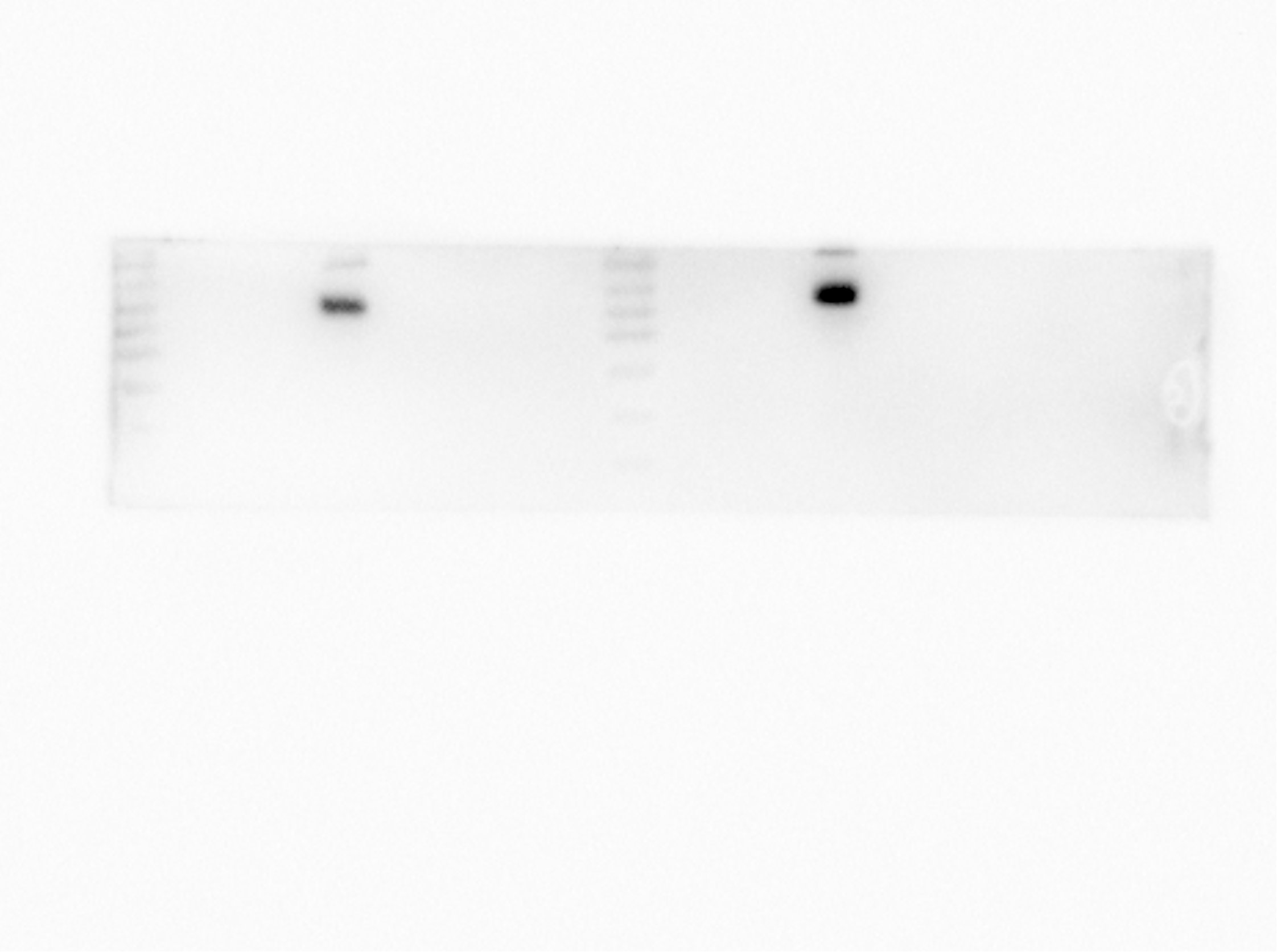

Supplement: S8 File — (ZIP) [file pone.0328981.s008.zip › RIPK3-2 (Anti-ASC).tif]
